# Supplementary material for: An Interactive Allyship and Privilege Workshop for Trainees in Medicine
Source: MedEdPORTAL. 2024 Aug 2;20:11426. doi: 10.15766/mep_2374-8265.11426 (PMC11294452; doi:10.15766/mep_2374-8265.11426)
Supplement: Supplementary file 1 — DEI Needs Assessment and Preworkshop Survey.docxFacilitator Guide.docxLearner Guide.docxAllyship Workshop Slides.pptxReflective Exercise.docxPostworkshop Survey.docx [file mep_2374-8265.11426-s001.zip › C. Learner Guide.docx]

**Allyship Workshop**

Learner’s Guide

Session Dates: [*Insert dates]*

Session Duration: 2hrs

Learners:

- [*Insert learners here]*

Faculty: Small group facilitators

Format: 2-hour group session with small breakout groups

## Session Overview:

In this 2-hour session, learners will be presented current data on unconscious bias seen within residency programs and perform self-reflective small group exercises.

## Curriculum Objectives

By the end of these sessions, learners will be able to:

1. Explain two key examples of allyship that can be demonstrated in your training or specialty in the reflection exercise.
2. Distinguish two key differences between *performative* allyship and *true* allyship in a small group activity.
3. Self-reflect on one’s own broad spectrum of privilege by identifying at least 2 privileges one holds in a small group exercise.
4. Develop an action plan for sponsoring or mentoring that mirrors the concept of allyship in the reflection exercise.
5. Develop as allies and accomplices in equity by practicing conversations around discrimination through the role model exercise.

## Pre-Session Preparation

**Recommended reading prior to workshop:**

- Atcheson S. Allyship – The key to unlocking the power of diversity. *Forbes* <https://www.forbes.com/sites/shereeatcheson/2018/11/30/allyship-the-key-to-unlocking-the-power-of-diversity/#5eed122349c6>
- Brown KT. Perceiving allies from the perspective of non-dominant group members: Comparisons to Friends and Activists. *Current Psychology* 2015;34:713-722. <https://doi.org/10.1007/s12144-014-9284-8>
- Ostrove JM, Brown KT. Are allies who we think they are?: A comparative analysis. *Journal of Applied Social Psychology* 2018;48(4):195-204. <https://onlinelibrary.wiley.com/doi/full/10.1111/jasp.12502>
