# Supplementary material for: An Interactive Allyship and Privilege Workshop for Trainees in Medicine
Source: MedEdPORTAL. 2024 Aug 2;20:11426. doi: 10.15766/mep_2374-8265.11426 (PMC11294452; doi:10.15766/mep_2374-8265.11426)
Supplement: Supplementary file 1 — DEI Needs Assessment and Preworkshop Survey.docxFacilitator Guide.docxLearner Guide.docxAllyship Workshop Slides.pptxReflective Exercise.docxPostworkshop Survey.docx [file mep_2374-8265.11426-s001.zip › E. Reflective Exercise.docx]

Reflective Exercise – Allyship in Practice

Survey Flow

Start of Block: Default Question Block

List two key examples of allyship that can be demonstrated in your training or specialty.

________________________________________________________________

End of Block: Default Question Block

Reflective Exercise - Sponsorship/Mentorship

Survey Flow

Start of Block: Default Question Block

What actions can we take in sponsoring or mentoring that mirrors the concept of allyship?

________________________________________________________________

End of Block: Default Question Block
