# Supplementary material for: An Interactive Allyship and Privilege Workshop for Trainees in Medicine
Source: MedEdPORTAL. 2024 Aug 2;20:11426. doi: 10.15766/mep_2374-8265.11426 (PMC11294452; doi:10.15766/mep_2374-8265.11426)
Supplement: Supplementary file 1 — DEI Needs Assessment and Preworkshop Survey.docxFacilitator Guide.docxLearner Guide.docxAllyship Workshop Slides.pptxReflective Exercise.docxPostworkshop Survey.docx [file mep_2374-8265.11426-s001.zip › A. DEI Needs Assessment and Preworkshop Survey.docx]

**DEI Needs Assessment**

**Start of Block: Default Question Block**

1 Create and enter an anonymous ID: 
1. First 3 letters of your current address
2. Last 2 letters of birth city
3. Last digit of year of birth
4. Number of siblings (not including self)
Example: Red, San Francisco, 1991, 3 brothers = REDCO13

________________________________________________________________

1. How strongly do you agree with the following?

|  | Strongly Disagree (1) | Somewhat Disagree (2) | Neutral (3) | Somewhat Agree (4) | Strongly Agree (5) |
| --- | --- | --- | --- | --- | --- |
| I know how to define the term *allyship*. (1) |  |  |  |  |  |
| As an ally, I am likely to mentor individuals that belong to a marginalized group and recommend them for other academic projects. (2) |  |  |  |  |  |
| I recognize the privilege that I have. (4) |  |  |  |  |  |

2. The following True/False questions are meant to evaluate your general understanding of the terms, and not what you would do personally.

3. Performative allyship is when someone from a non-marginalized group professes solidarity with a marginalized group in a way that is not helpful to that marginalized group.

- True (1)
- False (2)
- I'm not sure (3)

4. Please answer the following 4 questions in the context of life in general, and not just in work settings.

5.. Choose the response that is most applicable to you up to this point:

- I **talk** to others who look like me. (1)
- I **listen** to others who look differently than me. (2)
- I **socialize** with others who look differently than me. (3)

6. Choose the response that is most applicable to you up to this point:

- I **talk** to others who think like me. (1)
- I **listen** to others who think differently than me. (2)
- I **socialize** with others who think differently than me. (3)

7. Choose the response that is most applicable to you up to this point:

- I strive to be comfortable and don't usually address my own biases. (1)
- I understand my own biases and knowledge gaps and share them with others. (2)
- I don't let mistakes from my own biases deter me from continuing to critically evaluate my own biases. (3)

8. Choose the response that is most applicable to you at this point:

- I **avoid** hard questions about privilege and racism. (1)
- I **understand** my own privilege in ignoring racism. (2)
- I **speak out** when I see racism in action. (3)

9.. Have you received formal Diversity, Equity, and Inclusion (DEI) training before?

- Yes (1)
- No (2)

*Display This Question:*

*If Have you received formal Diversity, Equity, and Inclusion (DEI) training before? = Yes*

10. What was most effective in that DEI training?

________________________________________________________________

11. What does an ideal DEI training look like for you?

________________________________________________________________

12. Anything else you'd like to share?

________________________________________________________________

**End of Block: Default Question Block**

**Start of Block: Demographics**

13. Options for identities are abbreviated to preserve survey-takers' anonymity.

14. What department are you in?

- Anesthesia (1)
- Surgery (2)
- Prefer Not to Answer (3)

*Display This Question:*

*If What department are you in? = Anesthesia*

15. Which year of anesthesia postgraduate training are you?

- Intern (1)
- CA-1 (2)
- CA-2 (3)
- CA-3 (4)
- Fellow (5)
- Prefer not to answer (6)

*Display This Question:*

*If What department are you in? = Surgery*

16. Which year of surgery postgraduate training are you in?

- PGY-1 (1)
- PGY-2 (2)
- PGY-3 (3)
- Research Fellow (4)
- PGY-4 (5)
- PGY-5 (6)
- Prefer Not to Answer (7)

17. Race/ethnicity: (Choose all that apply)

- White/Caucasian (1)
- Black/African American (2)
- American Indian and/or Alaska Native (3)
- Asian (4)
- Native Hawaiian and/or other Pacific Islander (5)
- Latinx (6)
- Other (7) ________________________________________________
- Prefer not to answer (8)

18. Do you identify as Underrepresented In Medicine as defined by *[INSTITUTION NAME]*? 

 *Includes: [INSERT INSTITUTION’S DEFINITION OF UIM]*

- Yes (1)
- No (2)
- Prefer not to answer (3)

19. Gender Identity: Choose all that apply.

- Male (1)
- Female (2)
- Non-binary (3)
- Transgender (4)
- Prefer to Self-Describe (5) ________________________________________________
- Prefer Not to Answer (6)

20. Do you identify as a member of the LGBTQIA+ Community?

- Yes (1)
- No (2)
- Prefer not to answer (3)

**End of Block: Demographics**

Adapted from validated study:

Hu Y-Y, Ellis RJ, Hewitt DB, et al. Discrimination, Abuse, Harassment, and Burnout in Surgical Residency Training. *N Engl J Med*. 2019;381(18):1741-1752. doi:10.1056/NEJMsa1903759.
