# Supplementary material for: An Interactive Allyship and Privilege Workshop for Trainees in Medicine
Source: MedEdPORTAL. 2024 Aug 2;20:11426. doi: 10.15766/mep_2374-8265.11426 (PMC11294452; doi:10.15766/mep_2374-8265.11426)
Supplement: Supplementary file 1 — DEI Needs Assessment and Preworkshop Survey.docxFacilitator Guide.docxLearner Guide.docxAllyship Workshop Slides.pptxReflective Exercise.docxPostworkshop Survey.docx [file mep_2374-8265.11426-s001.zip › B. Facilitator Guide.docx]

**Allyship Workshop**

Faculty Facilitator’s Guide

Session Dates: *[Insert dates]*

Session Duration: 2hrs

Learners:

- [*Insert learner groups here]*

Faculty: Small group facilitators

Format: 2-hour group session with small breakout groups

## Session Overview:

In this 2-hour session, learners will be presented current data on unconscious bias seen within residency programs and perform self-reflective small group exercises.

## Curriculum Objectives

By the end of these sessions, learners will be able to:

1. Explain two key examples of allyship that can be demonstrated in your training or specialty in the reflection exercise.
2. Distinguish two key differences between *performative* allyship and *true* allyship in a small group activity.
3. Self-reflect on one’s own broad spectrum of privilege by identifying at least 2 privileges one holds in a small group exercise.
4. Develop an action plan for sponsoring or mentoring that mirrors the concept of allyship in the reflection exercise.
5. Develop as allies and accomplices in equity by practicing conversations around discrimination through the role model exercise.

## Pre-Session Preparation

**Recommended reading prior to workshop:**

- Atcheson S. Allyship – The key to unlocking the power of diversity. *Forbes*
- Brown KT. Perceiving allies from the perspective of non-dominant group members: Comparisons to Friends and Activists. *Current Psychology* 2015;34:713-722.
- Ostrove JM, Brown KT. Are allies who we think they are?: A comparative analysis. *Journal of Applied Social Psychology* 2018;48(4):195-204.

**Prior to this session facilitators should:**

- Review the Circles of My Multicultural Exercise (slide 6, Appendix D)
- Review the Privilege Wheel and Social Identity worksheet (slides 17-18, Appendix D)

## Session Outline

| **Duration** | **Topic** | **Who** |
| --- | --- | --- |
| 10 min | Introduction of Large Group / Goals and Objectives | Facilitator and Learners |
| 7 min | Introduction within Small Group: During these challenging times, what’s been a recent success? | Small Group of 5 learners + 1 facilitator |
| 20 min | Circles of My Multicultural Self | Small Group of 5 learners + 1 facilitator |
| 3 min | Large group debrief | Facilitator and Learners |
| 10 min | Brief didactic on Allyship, Power, and Privilege, Sponsoring, Mentoring | Facilitator and Learners |
| 3 min | Reflection Exercise | Learners |
| 5 min | Privilege wheel explanation | Facilitator and Learners |
| 20 min | Privilege wheel completion and Small group debrief | Small Group of 5 learners + 1 facilitator |
| 5 min | Large group debrief | Facilitator and Learners |
| 3 min | Reflection Exercise | Learners |
| 2 min | Preparation Exercise – Demonstration First | Facilitator and Learners |
| 20 min | Preparation Exercise - Small Group Debrief | Small Group of 5 learners + 1 facilitator |
| 2 min | Large group debrief | Facilitator and Learners |
| 5 min | Online Post-workshop Survey | Learners |
| 5 min | Wrap Up: Share ideas for Diversity Curriculum | Facilitator and Leaners |

## Allyship Session

- Intro in a large group setting **(10 min)**
  - Name
  - Pronoun
  - Name one thing that you value from any previous session that you have had on equity and inclusion.
- Review goals and objectives and house rules as a large group
- Intro in a small group setting **(7 min)**
  - “During these challenging times, what’s been a recent success?”
- Circles of My Multicultural Self Exercise **(20 min)**
  - Small Group Instructions
    - As a small group facilitator, instruct the learners that they have 5 minutes to complete the exercise.
    - After 5min, please start debriefing as a small group.
    - Possible debrief questions:
      - Share a story about a time you were especially ***proud*** to identify with one of the descriptors you used above.
      - Share a story about a time it was especially ***challenging*** to be identified with one of your identifiers or descriptors ***that you feel comfortable talking about*.**
      - Name a stereotype associated with one of the groups with which you identify that is ***not consistent*** with who you are.
  - Large Group Debrief **(3 min)**
  - **Summary of exercise:** In Circles of My Multicultural Self, each participant selects 4 descriptors with which they most strongly identified. Then, they share one story about a time they were particularly proud to identify as one of the identities, another story about a time it was painful to identify with one of the identities, and name a stereotype associated with one of their identities that is not consistent with who they are. Participants are encouraged to not only look internally but to also think critically about the stereotypes they have applied to others and how to address those thoughts.
- Brief Didactic on Allyship, Power and Privilege **(10 min)**
- Reflection Exercise **(3 min)**
  - List two key examples of allyship that can be demonstrated in your training or specialty.
  - (Create Qualtrics survey or some other online survey link for this)
- Privilege Wheel Explanation **(5 min)**
- Privilege Wheel and Social Identity Exercise **(15 min)**
  - Give learners 5 minutes to complete their own personal privilege wheel in association with the social identity worksheet.
  - As a small group, the following debriefing questions will be asked by the assigned facilitator.
    - What surprised you?
    - How does your privilege affect how people see you?
    - What is your next step in using your power in being an anti-racist?
    - Any other questions or comments?
  - Large group debrief **(5 min)**
  - **Summary of exercise:** In the Privilege Wheel activity, adapted from a previously published exercise, participants are instructed to map out their different identities and share how they were perceived in different contexts.^11^ This has previously led to discussions around how privilege can normalize certain identities over others.
- Reflection exercise **(3 min)**
  - What actions can we take in sponsoring or mentoring that mirrors the concept of allyship?
  - (Create Qualtrics or some other online survey link for this)
- Preparation Exercise – Facilitators will first demonstrate with each other **(2 min)**
  - Speaker
    - “I wish that we had equity here all the time at this hospital. The reality is that unskillful or hurtful things may be said or heard related to identity – gender, race, religious identity, LGBTQIA identity. I’m going to check in with you and all members of the team regularly about experiences of bias and microaggression.”
  - Respondent
    - “What was effective in the way your partner said this.”
  - Breakout groups to practice preparation phrase with a partner **(20 min)**
    - Debrief how this could be used in real life.
    - Assign reporter
    - Each person should take turns and practice a preparation phrase within their small group.
  - Large Group Debrief **(2 min)**
  - **Summary of exercise:** The Preparation Exercise is a novel activity developed by one of our facilitators. The goal is to provide space for participants to practice effective strategies of approaching the topic of inequity and discrimination. These are topics that can be challenging to address in the workplace and therefore can often go unaddressed. Facilitators will first provide an example while in the large group (the written phrasing). Participants are then split into breakout rooms where they practice their own authentic phrases and reflected on how this can be applied in real life. Participants give each other feedback and share what they have find effective in the different ways that participants approached the conversation.
- Post-workshop Survey **(5 min)**
  - (Consider pasting link here so facilitators have access)
- Wrap Up **(5 min)**
  - Have residents share ideas for what they would like to see addressed in the diversity curriculum going forward.
  - Name one thing you are taking away with you.
  - Consider doing a word cloud here so participants can see popular themes
