# Supplementary material for: An Interactive Allyship and Privilege Workshop for Trainees in Medicine
Source: MedEdPORTAL. 2024 Aug 2;20:11426. doi: 10.15766/mep_2374-8265.11426 (PMC11294452; doi:10.15766/mep_2374-8265.11426)
Supplement: Supplementary file 1 — DEI Needs Assessment and Preworkshop Survey.docxFacilitator Guide.docxLearner Guide.docxAllyship Workshop Slides.pptxReflective Exercise.docxPostworkshop Survey.docx [file mep_2374-8265.11426-s001.zip › D. Allyship Workshop Slides.pptx]

## Slide 1
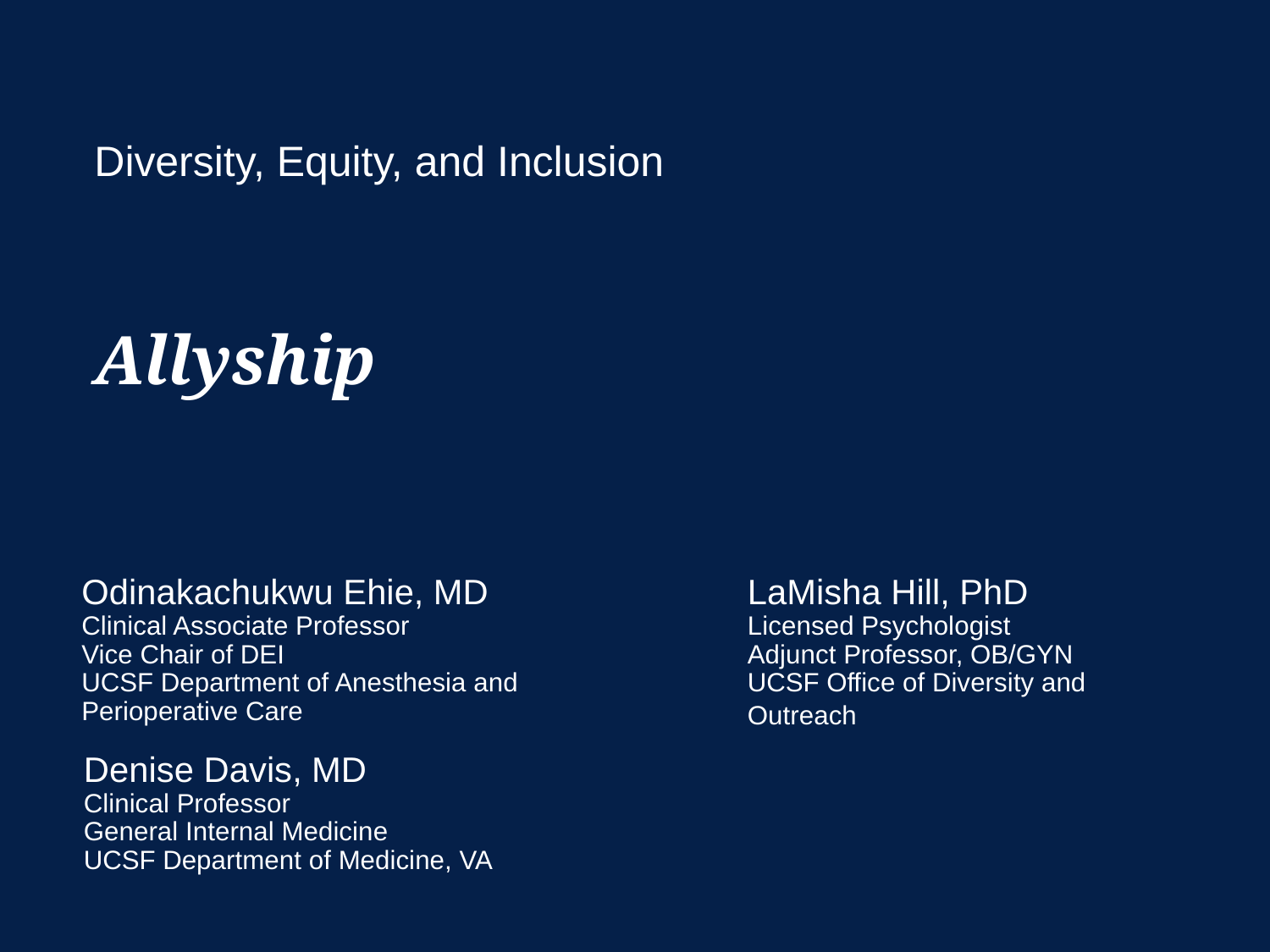

# Diversity, Equity, and Inclusion
Allyship
LaMisha Hill, PhD
Licensed Psychologist​
Adjunct Professor, OB/GYN​
UCSF Office of Diversity and Outreach​
Odinakachukwu Ehie, MD
Clinical Associate Professor
Vice Chair of DEI
UCSF Department of Anesthesia and Perioperative Care
Denise Davis, MD
Clinical Professor
General Internal Medicine
UCSF Department of Medicine, VA

## Slide 2
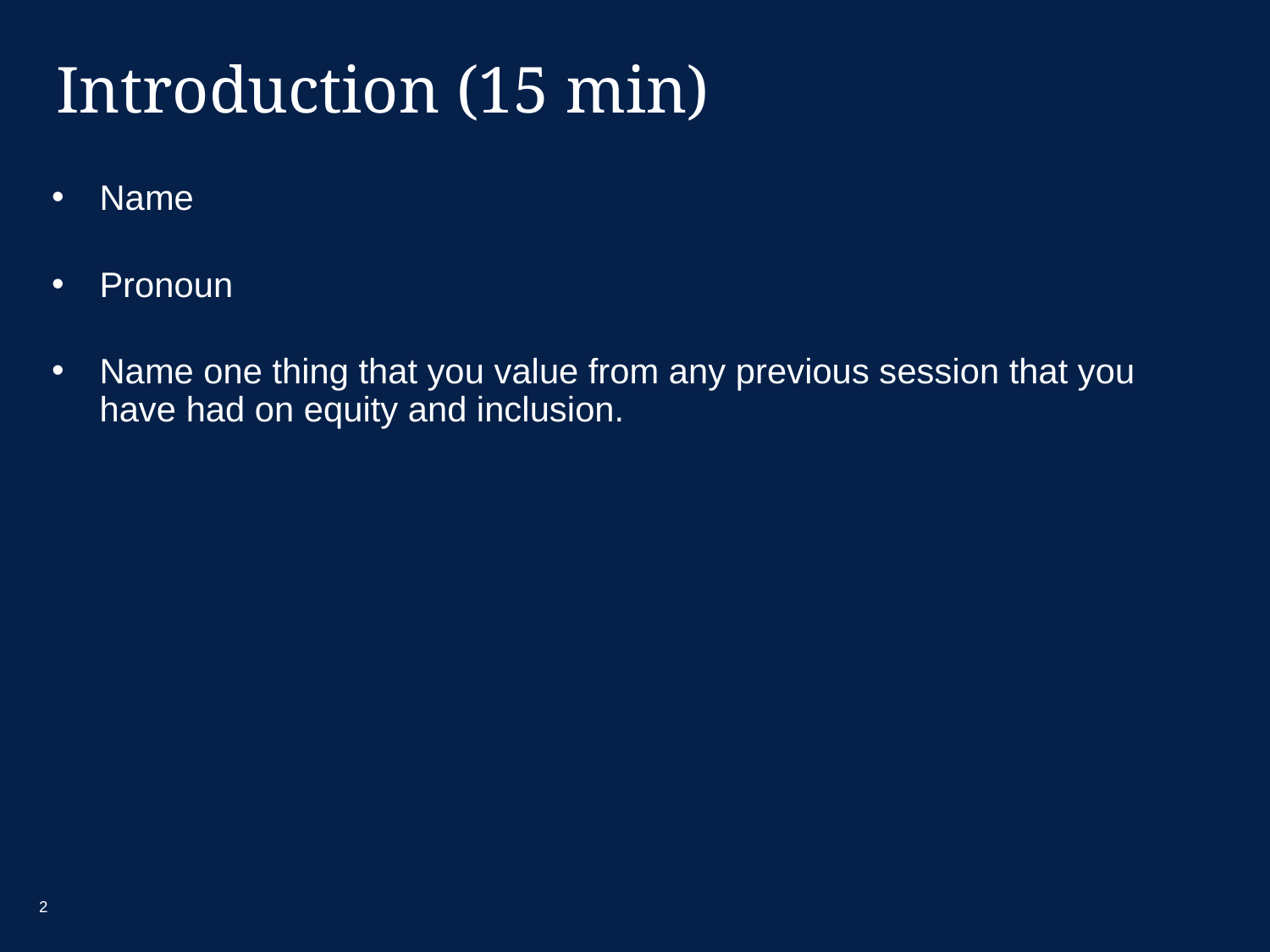

# Introduction (15 min)
Name
Pronoun
Name one thing that you value from any previous session that you have had on equity and inclusion.
1

## Slide 3
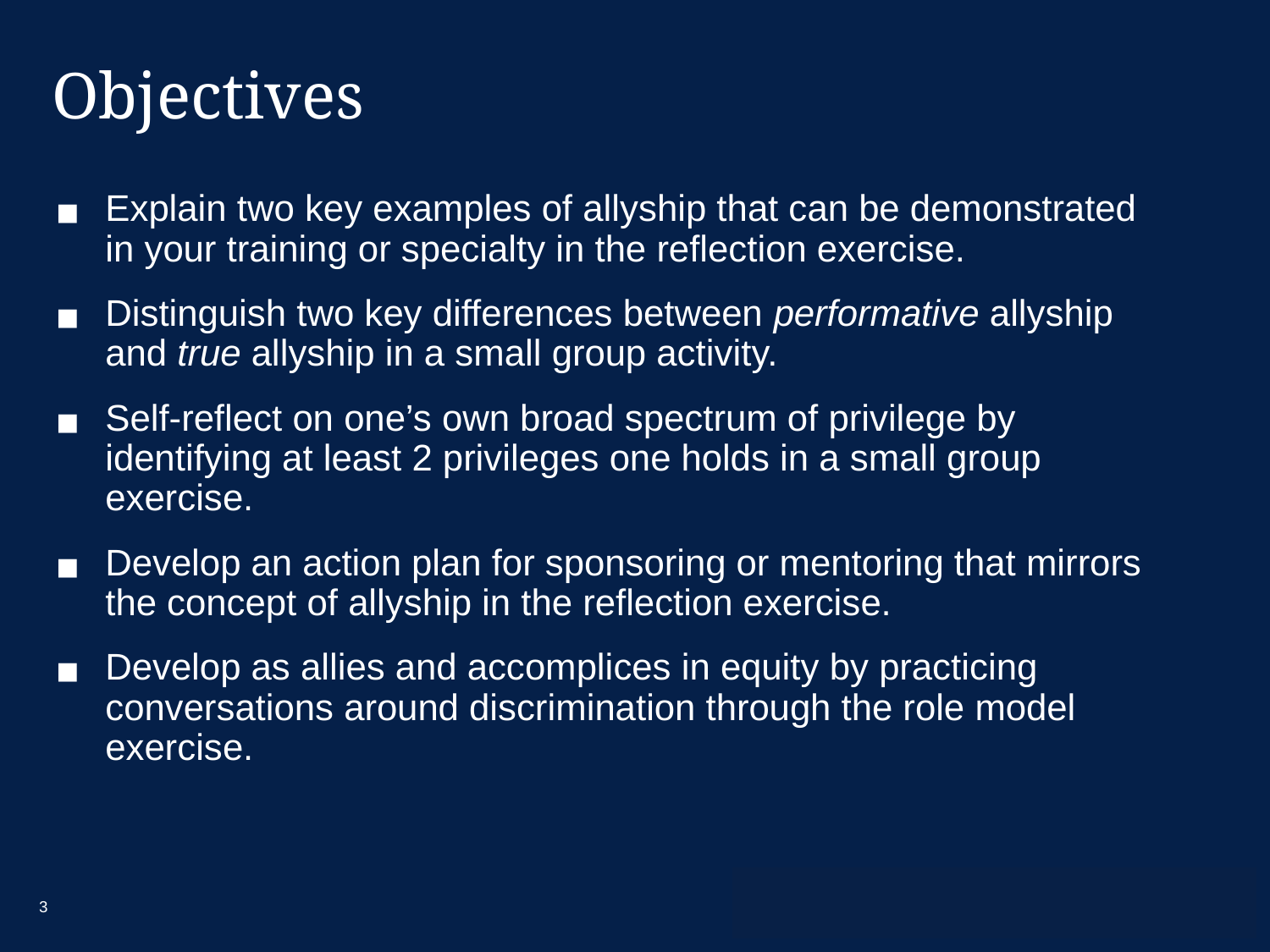

# Objectives
Explain two key examples of allyship that can be demonstrated in your training or specialty in the reflection exercise.
Distinguish two key differences between performative allyship and true allyship in a small group activity.
Self-reflect on one’s own broad spectrum of privilege by identifying at least 2 privileges one holds in a small group exercise.
Develop an action plan for sponsoring or mentoring that mirrors the concept of allyship in the reflection exercise.
Develop as allies and accomplices in equity by practicing conversations around discrimination through the role model exercise.
2

## Slide 4
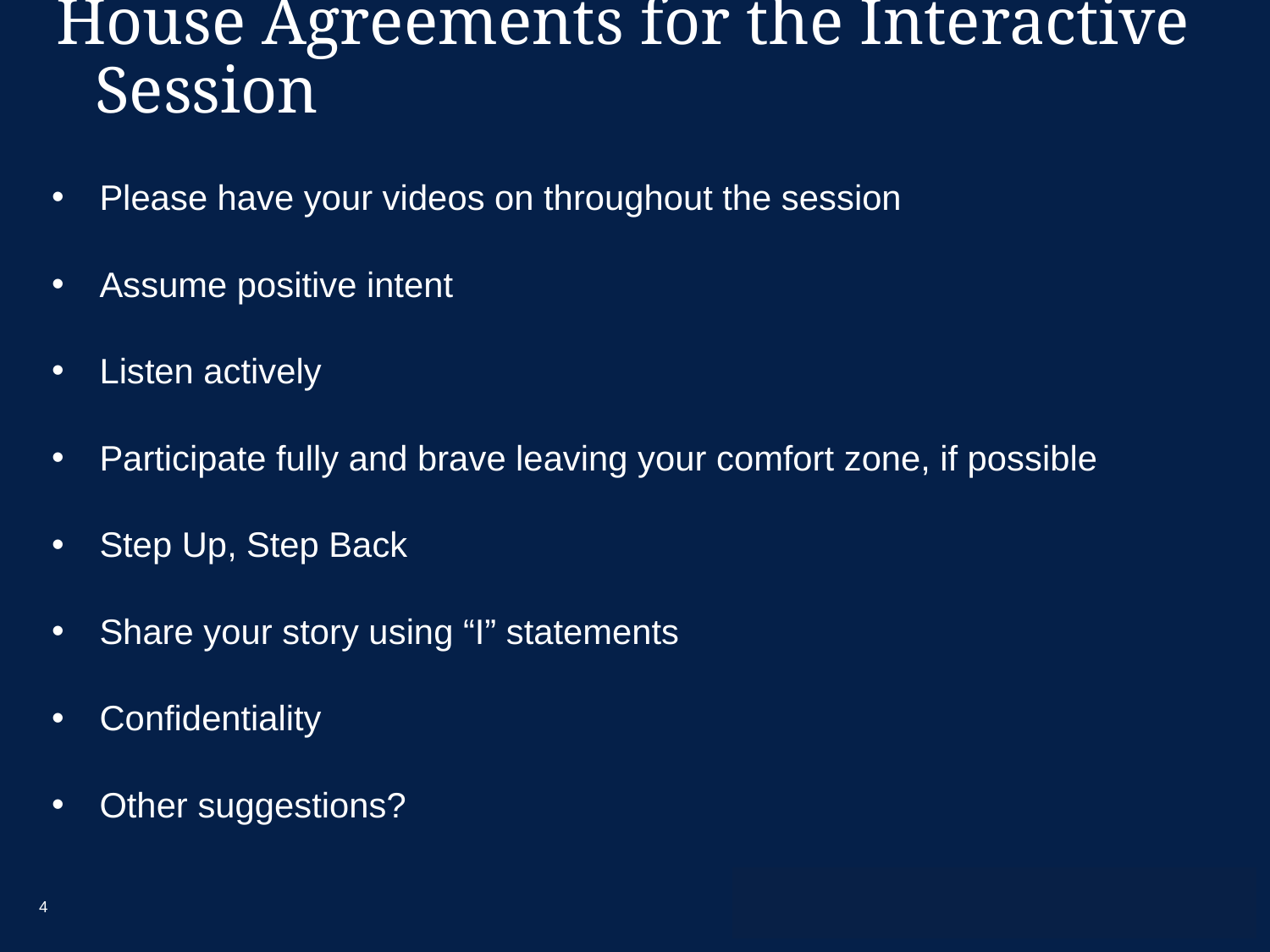

# House Agreements for the Interactive Session
Please have your videos on throughout the session
Assume positive intent
Listen actively
Participate fully and brave leaving your comfort zone, if possible
Step Up, Step Back
Share your story using “I” statements
Confidentiality
Other suggestions?
3

## Slide 5
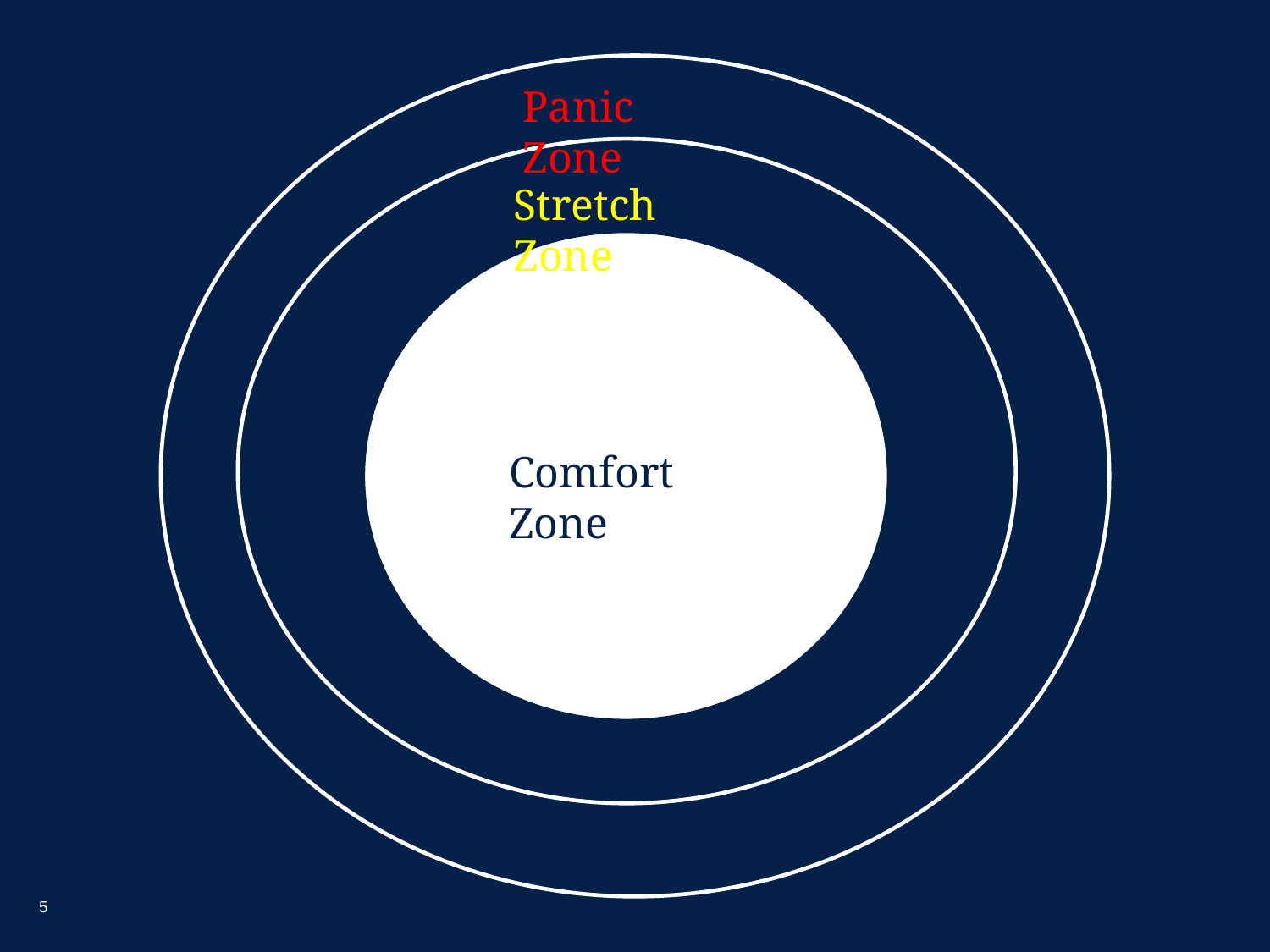

Panic Zone
Stretch Zone
Comfort Zone
4

## Slide 6
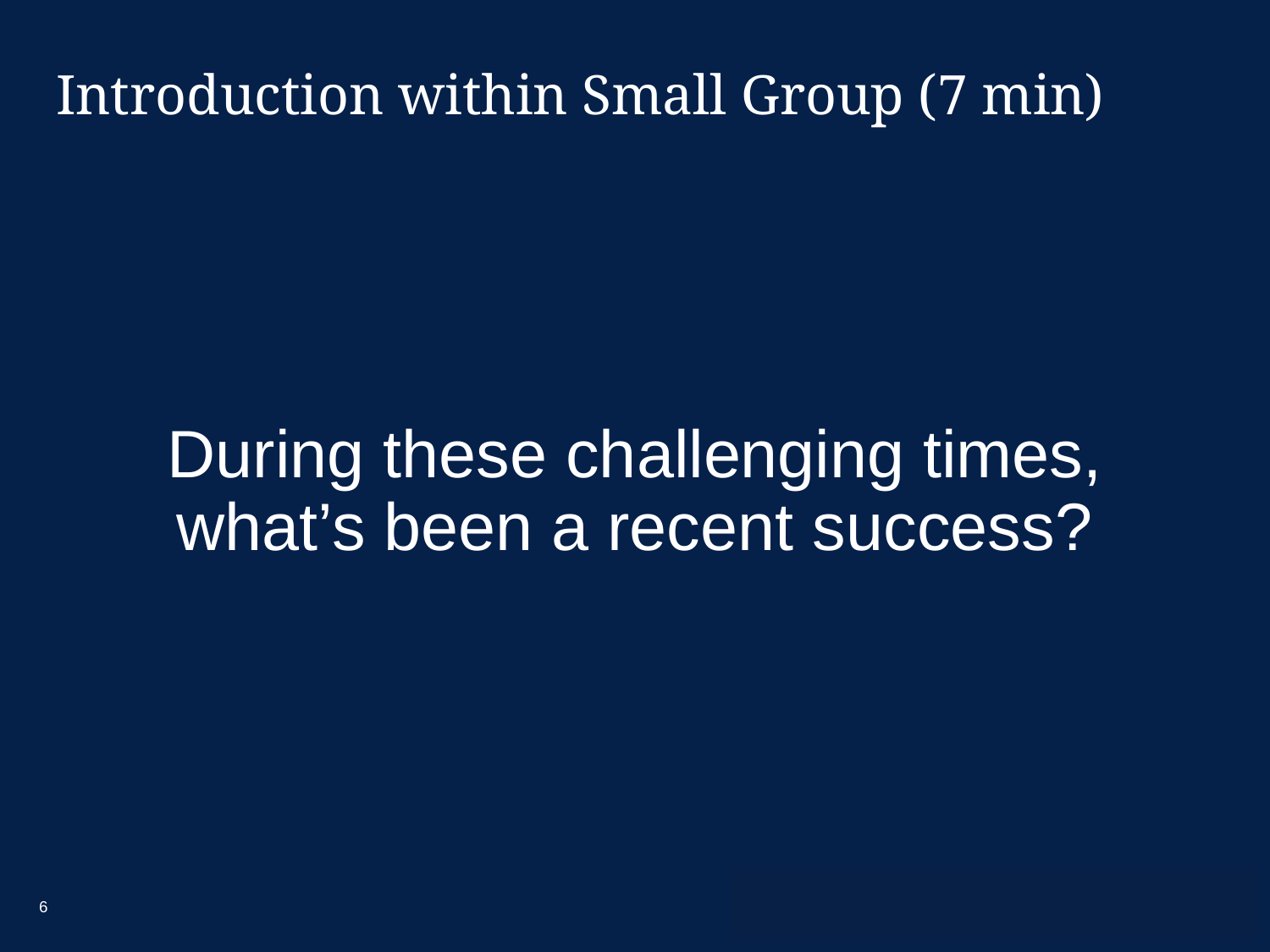

# Introduction within Small Group (7 min)
During these challenging times, what’s been a recent success?
5

## Slide 7
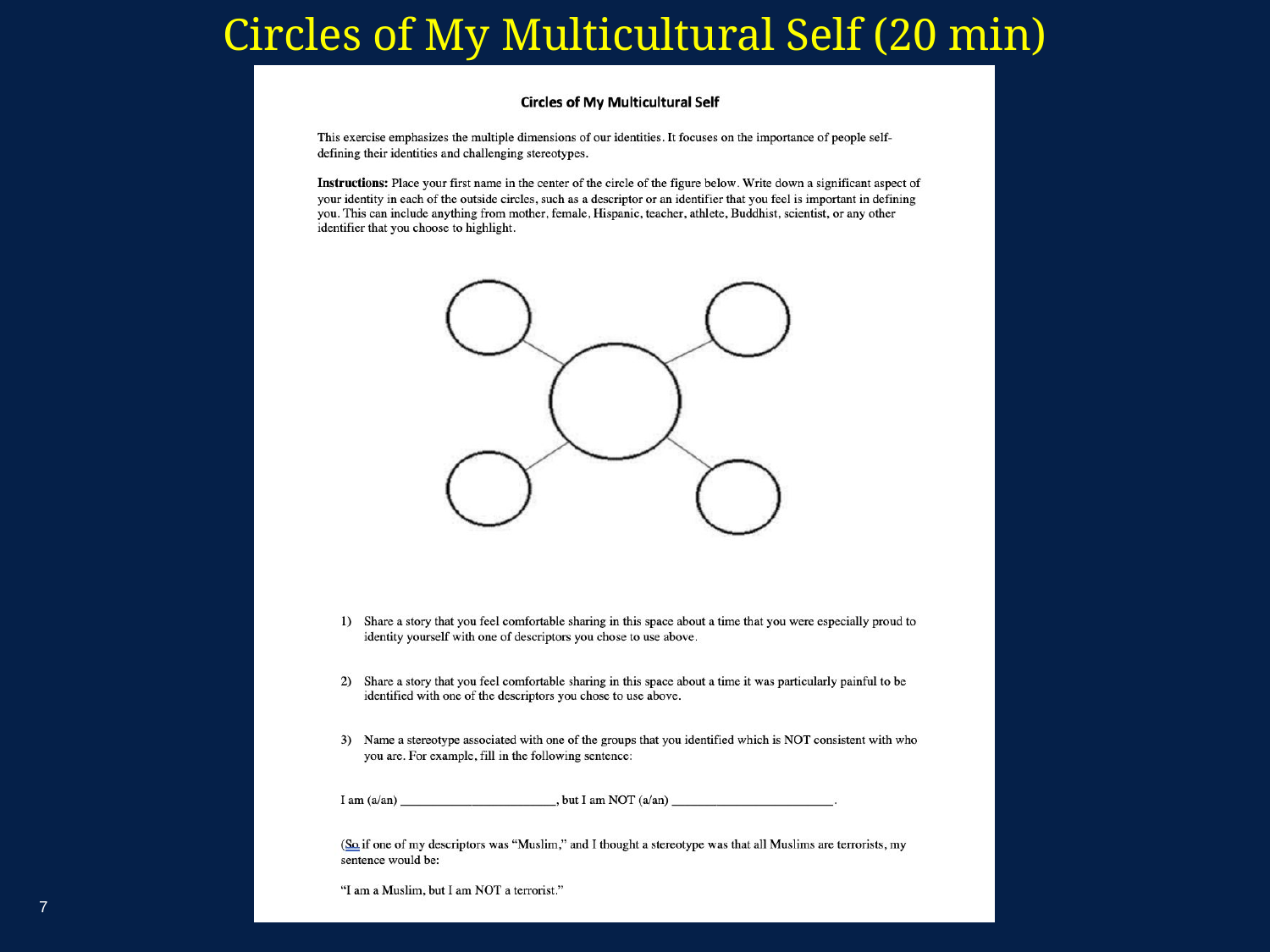

# Circles of My Multicultural Self (20 min)
Image adapted from: https://www.uh.edu/cdi/diversity_education/resources/_files/_activities/diversity-activities-resource-guide.pdf on Nov 13 2020. Permission received from Varselles Cummings (representative of the Center for Diversity and Inclusion at the University of Houston)
6

## Slide 8
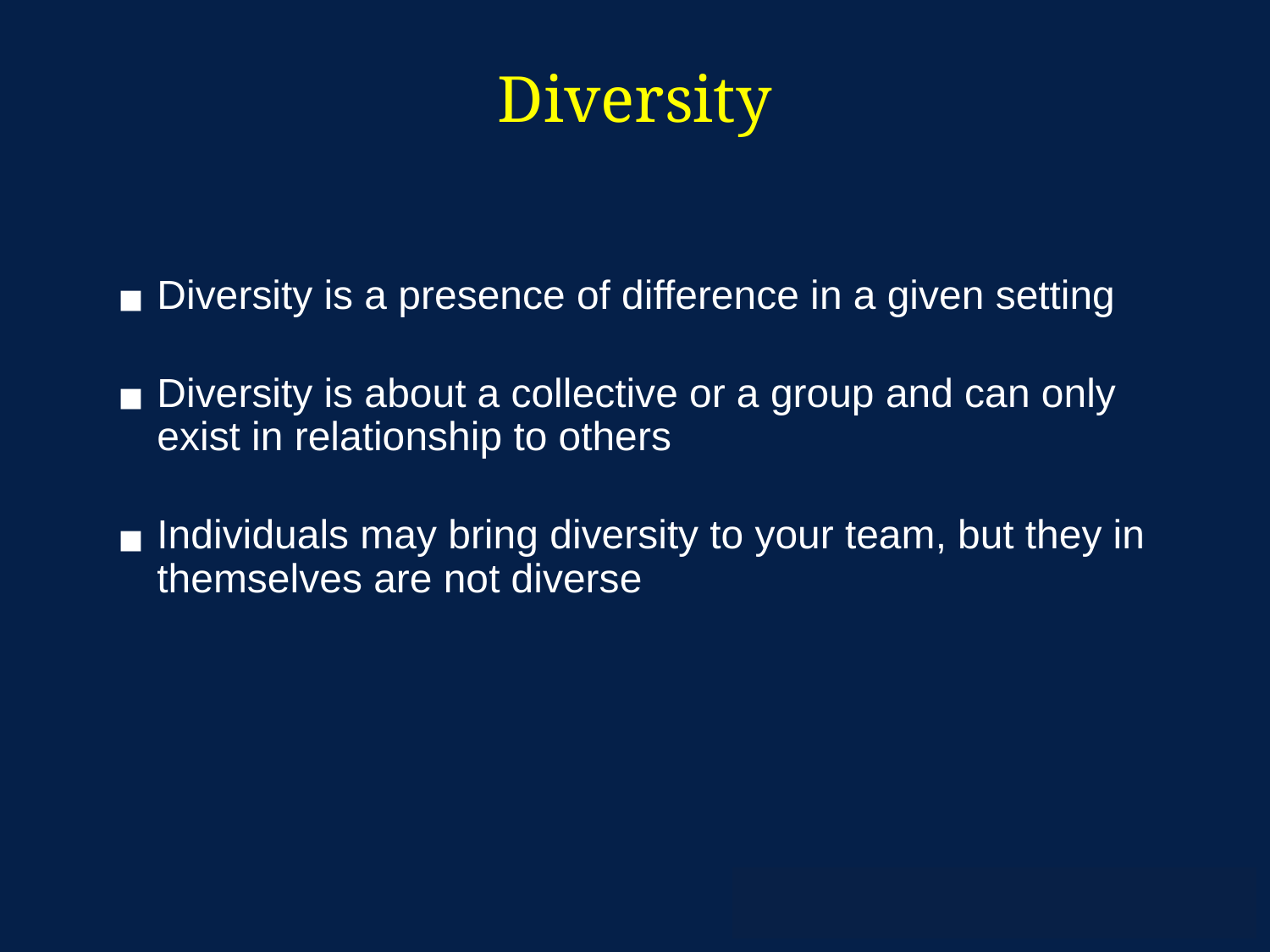

# Diversity
Diversity is a presence of difference in a given setting
Diversity is about a collective or a group and can only exist in relationship to others
Individuals may bring diversity to your team, but they in themselves are not diverse

## Slide 9
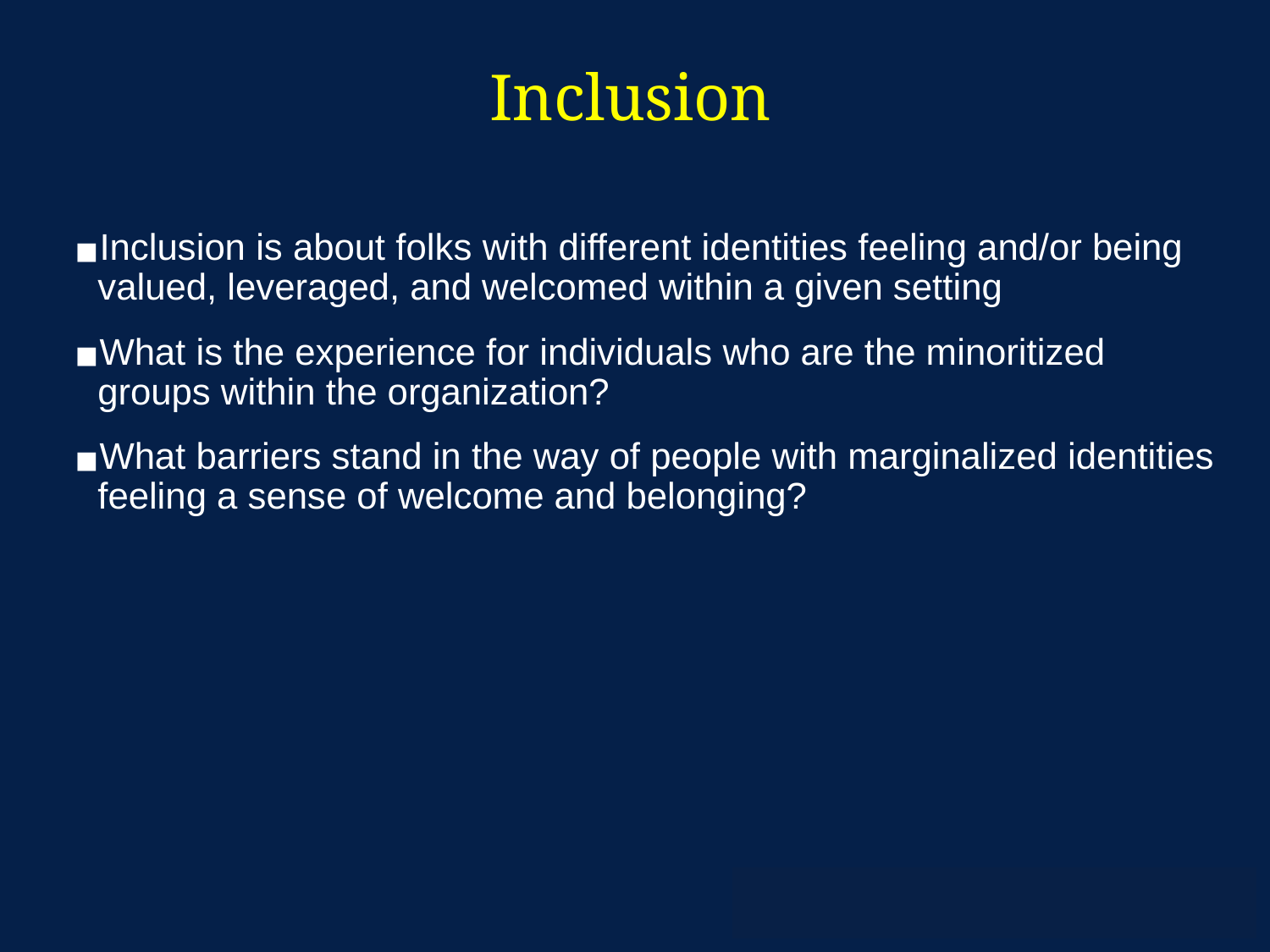

# Inclusion
Inclusion is about folks with different identities feeling and/or being valued, leveraged, and welcomed within a given setting
What is the experience for individuals who are the minoritized groups within the organization?
What barriers stand in the way of people with marginalized identities feeling a sense of welcome and belonging?

## Slide 10
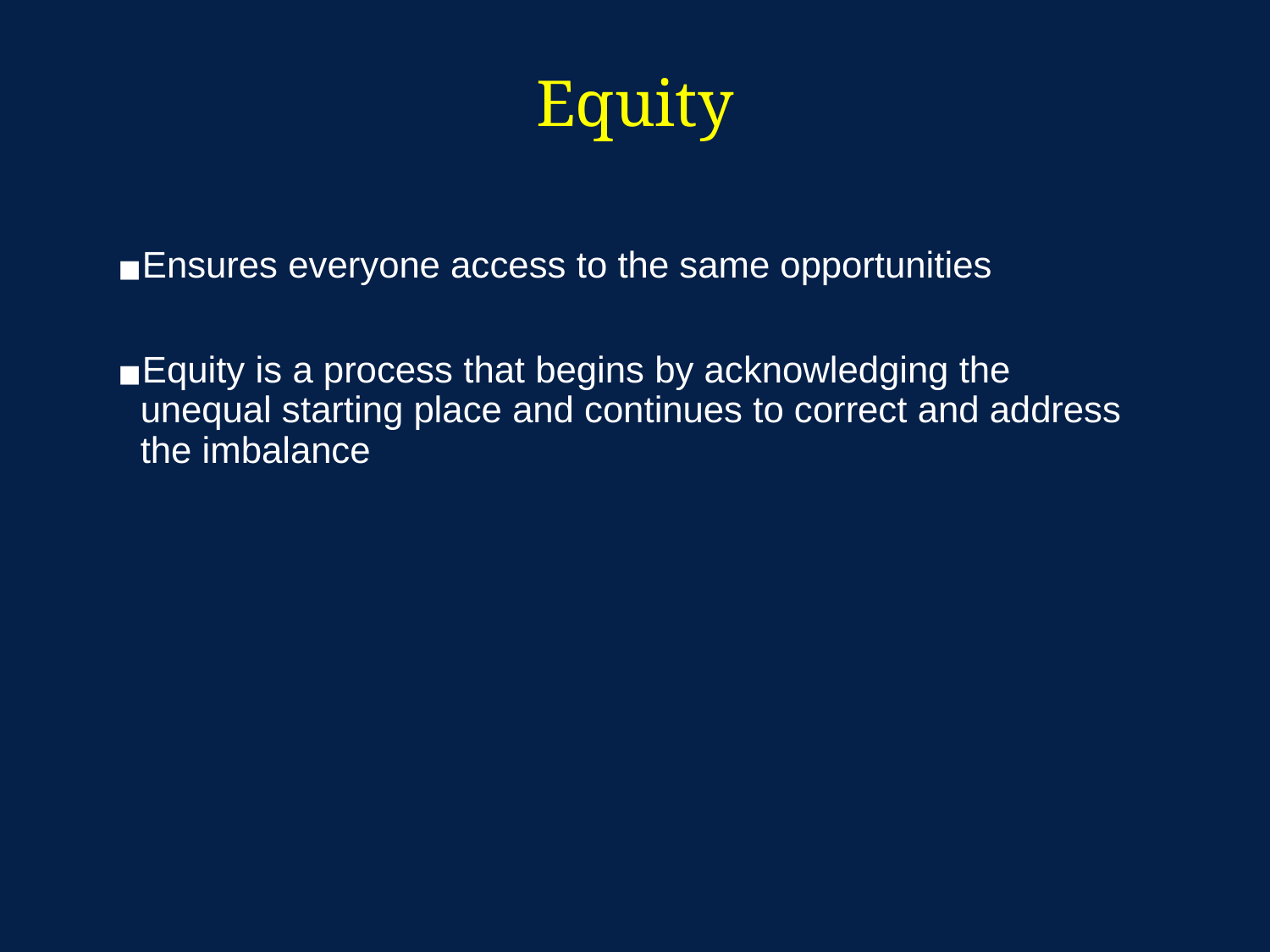

# Equity
Ensures everyone access to the same opportunities
Equity is a process that begins by acknowledging the unequal starting place and continues to correct and address the imbalance

## Slide 11
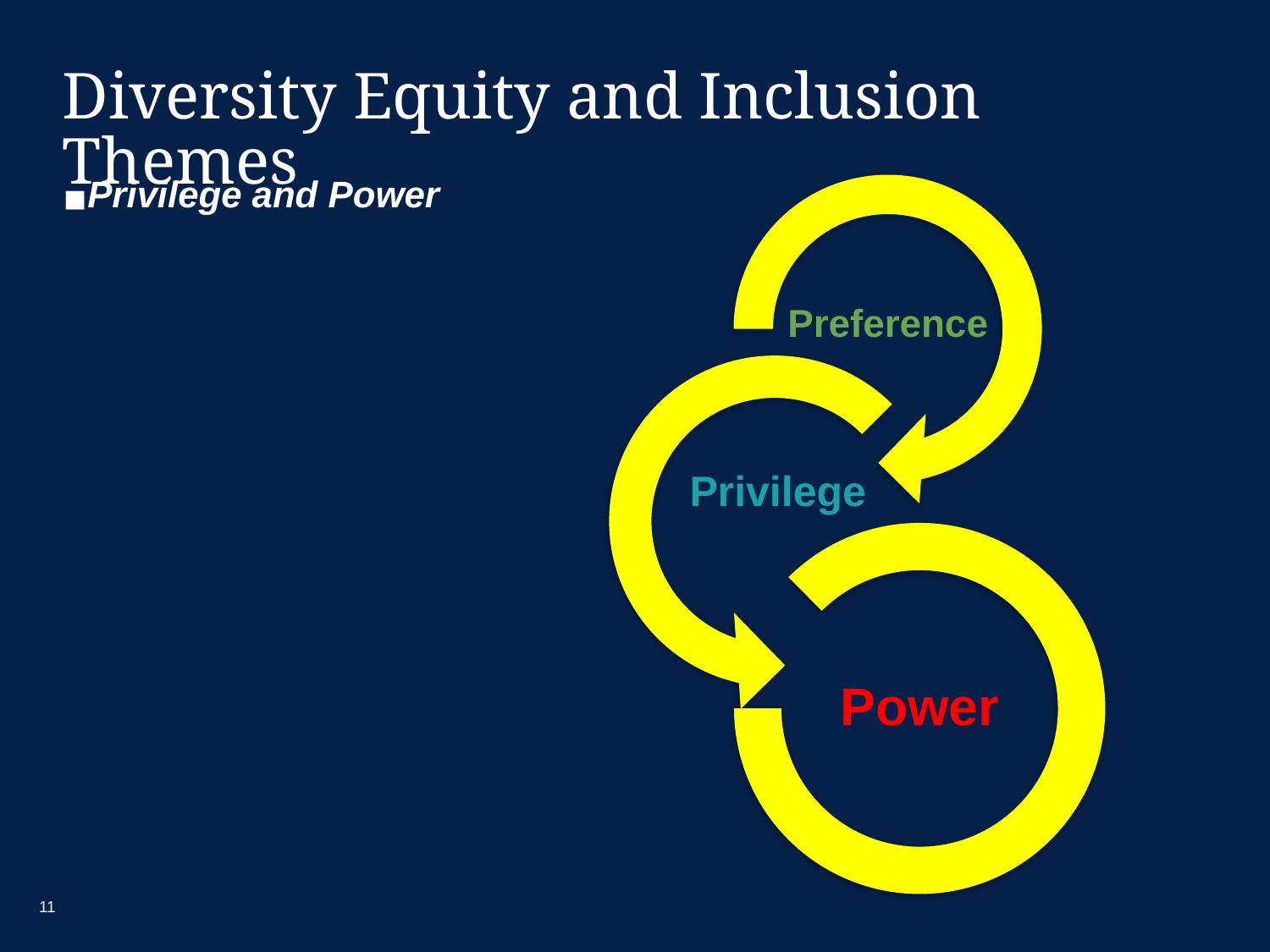

# Diversity Equity and Inclusion Themes
Preference
Privilege
Power
Privilege and Power
10

## Slide 12
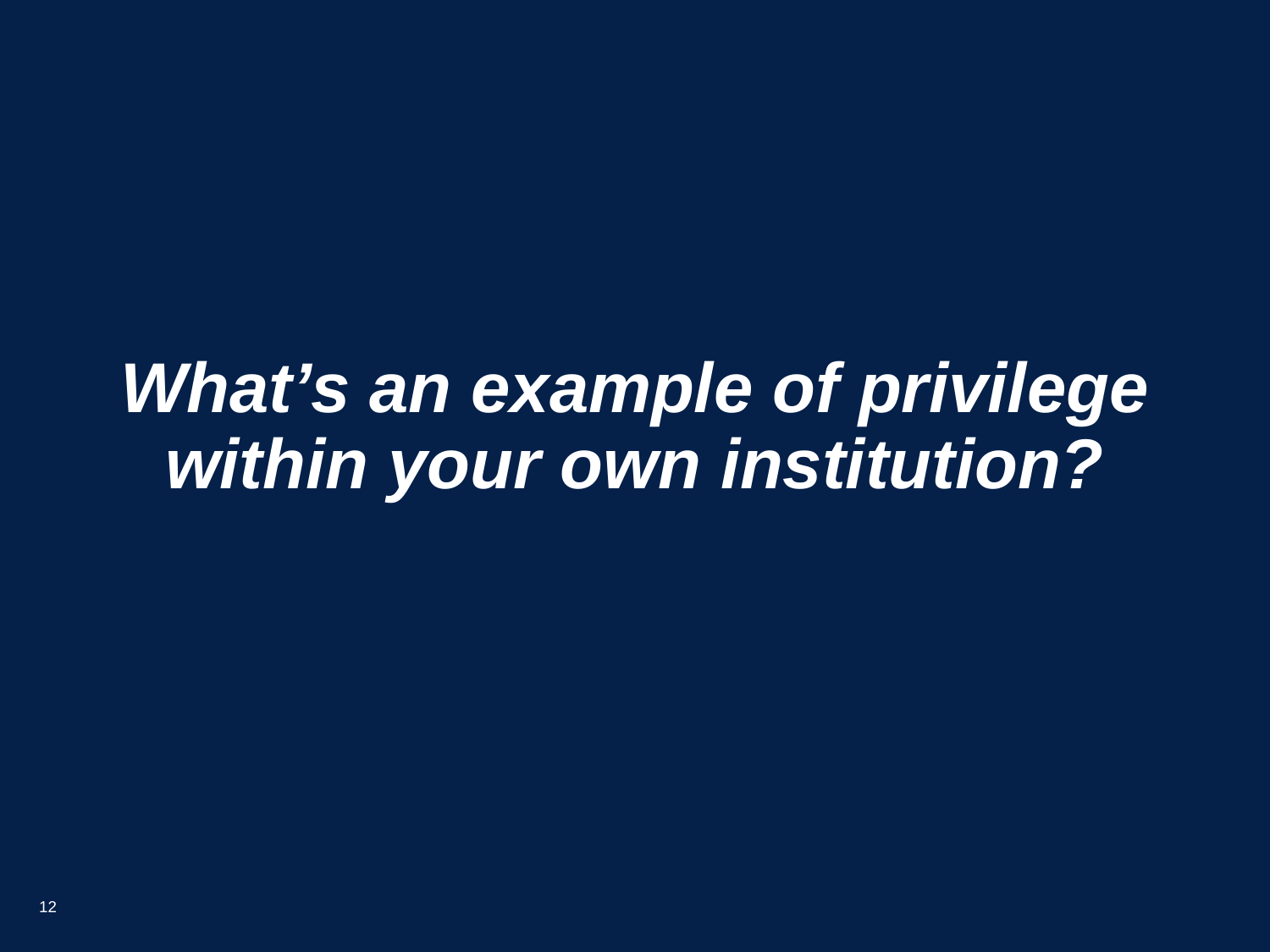

What’s an example of privilege within your own institution?
11

## Slide 13
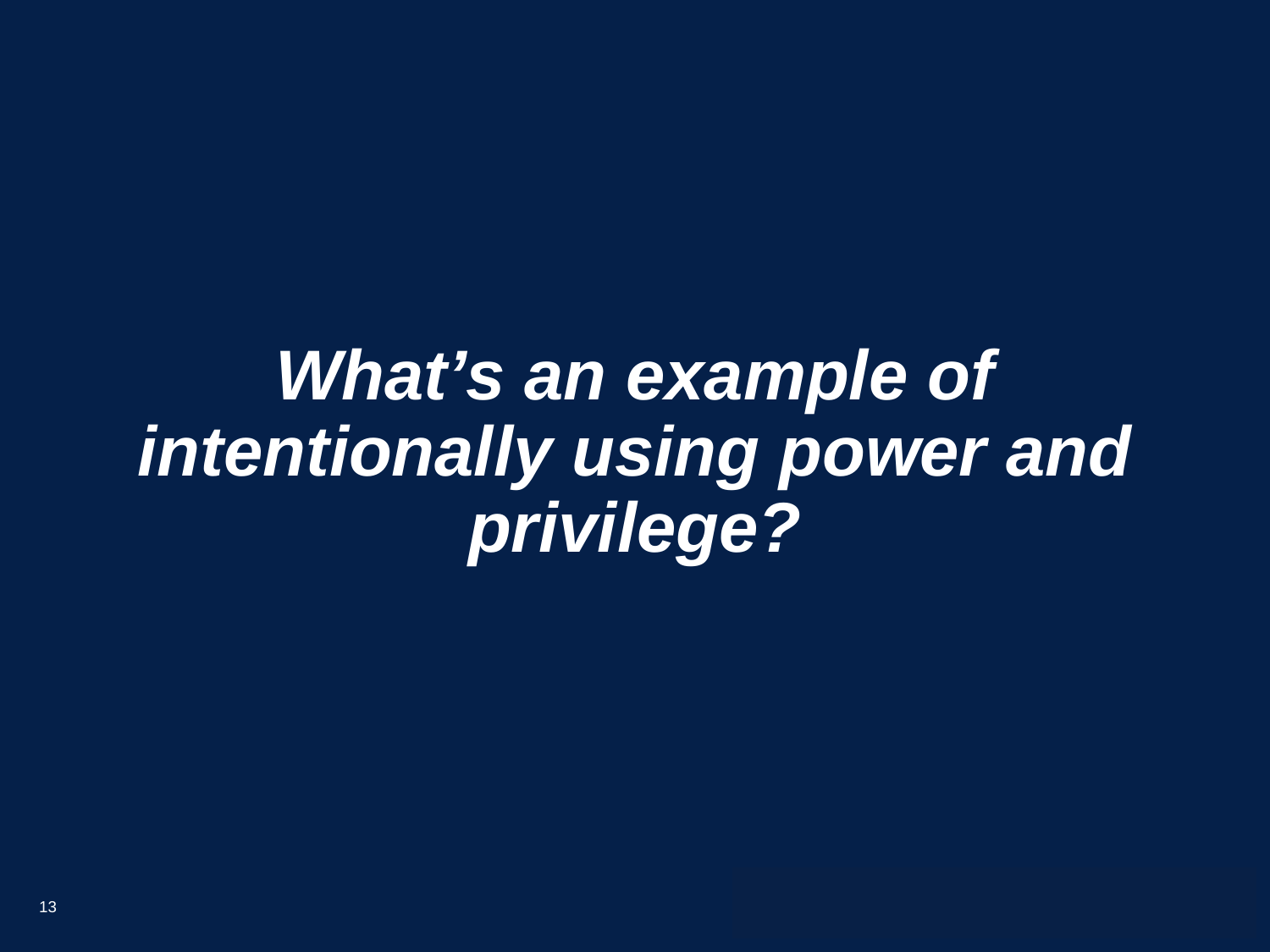

What’s an example of intentionally using power and privilege?
12

## Slide 14
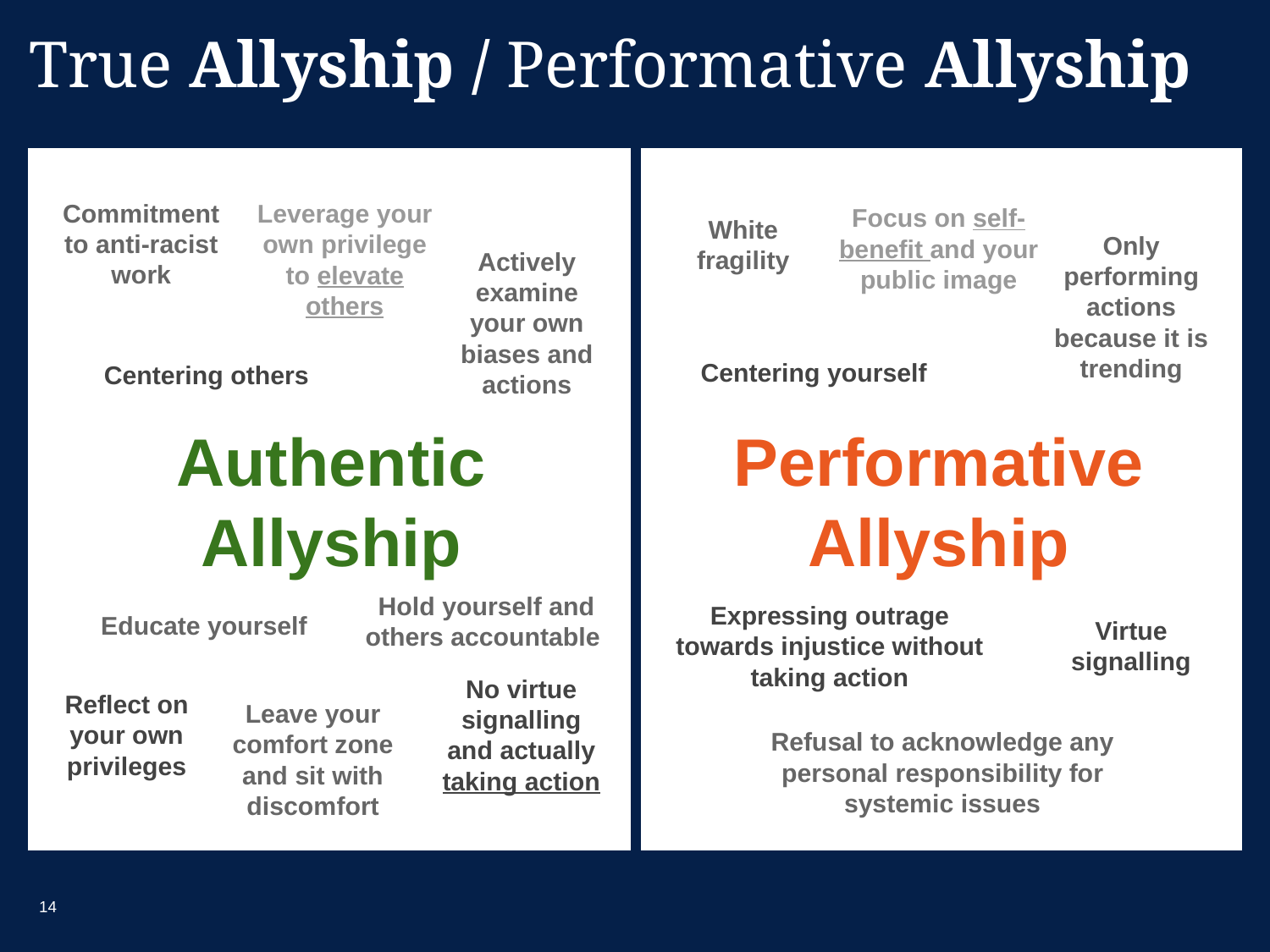

# True Allyship / Performative Allyship
Authentic Allyship
Commitment to anti-racist work
Leverage your own privilege to elevate others
Actively examine your own biases and actions
Centering others
Educate yourself
No virtue signalling and actually taking action
Reflect on your own privileges
Leave your comfort zone and sit with discomfort
Performative Allyship
Focus on self-benefit and your public image
White fragility
Only performing actions because it is trending
Centering yourself
Virtue signalling
Refusal to acknowledge any personal responsibility for systemic issues
Hold yourself and others accountable
Expressing outrage towards injustice without taking action
Refusal to acknowledge any personal responsibility
13

## Slide 15
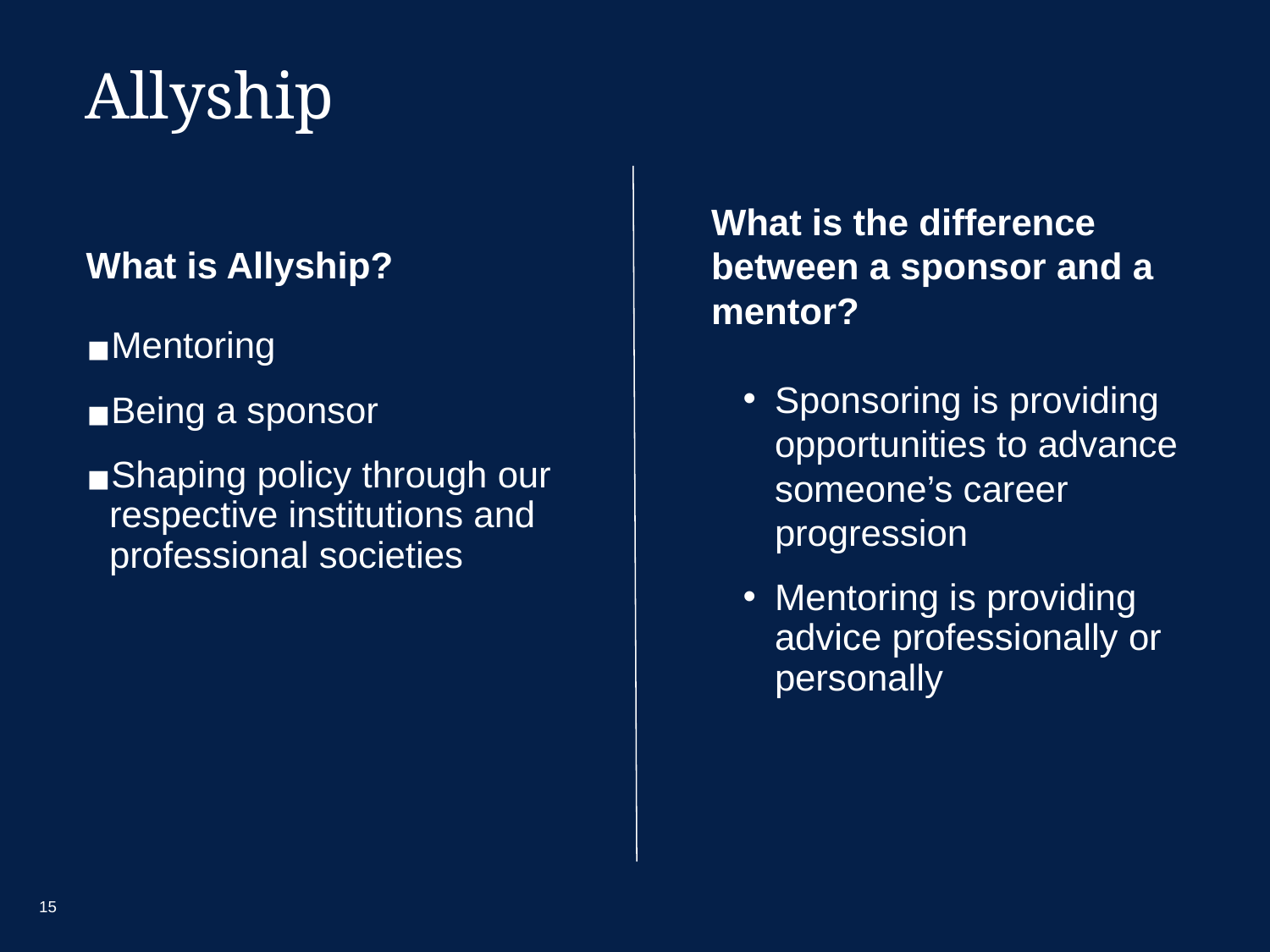

# Allyship
What is the difference between a sponsor and a mentor?
Sponsoring is providing opportunities to advance someone’s career progression
Mentoring is providing advice professionally or personally
What is Allyship?
Mentoring
Being a sponsor
Shaping policy through our respective institutions and professional societies
14

## Slide 16
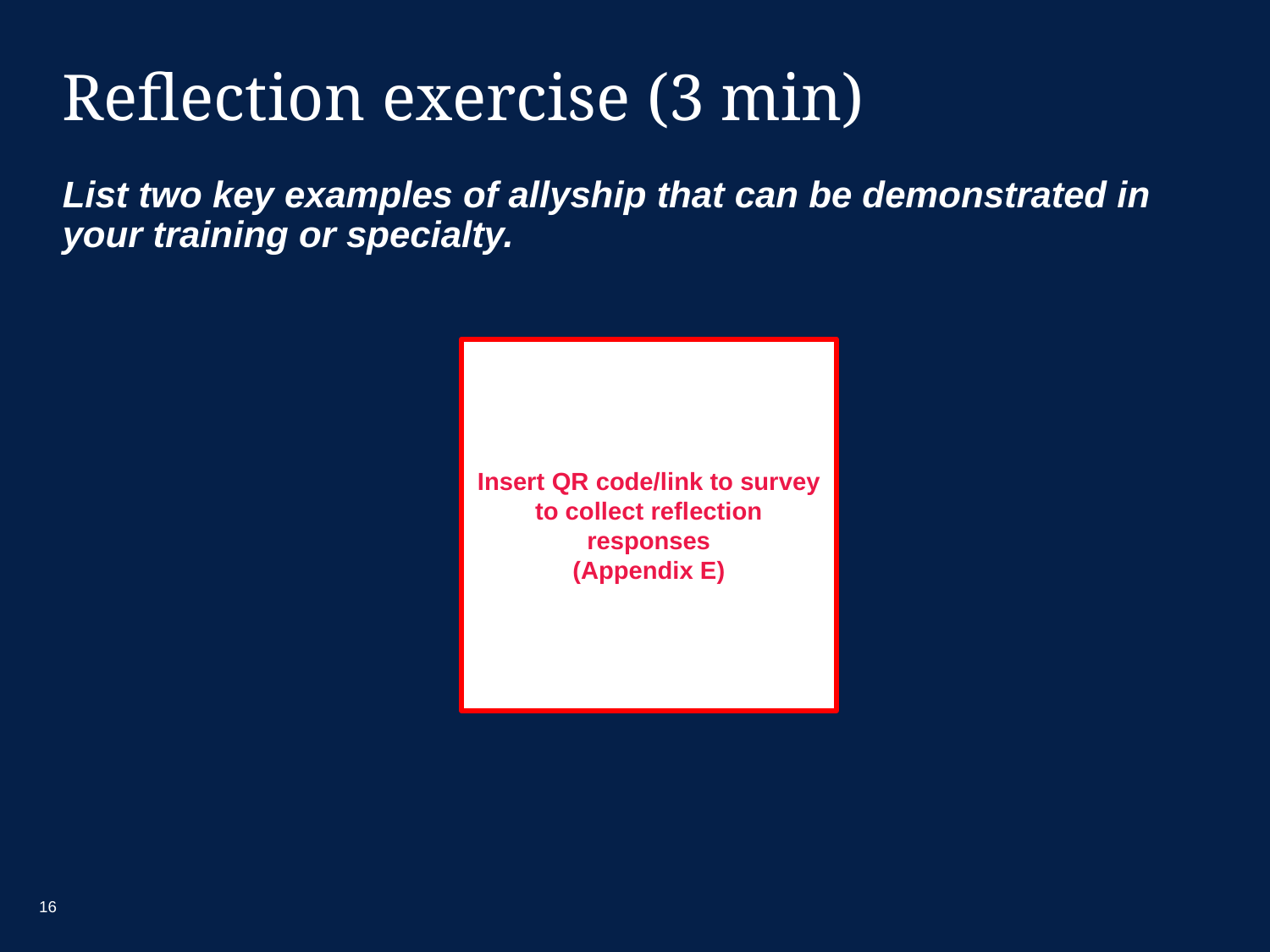

Reflection exercise (3 min)
List two key examples of allyship that can be demonstrated in your training or specialty.
Insert QR code/link to survey to collect reflection responses
(Appendix E)
https://ucsf.co1.qualtrics.com/jfe/form/SV_6Dsrpr5Va68SEu1
15

## Slide 17
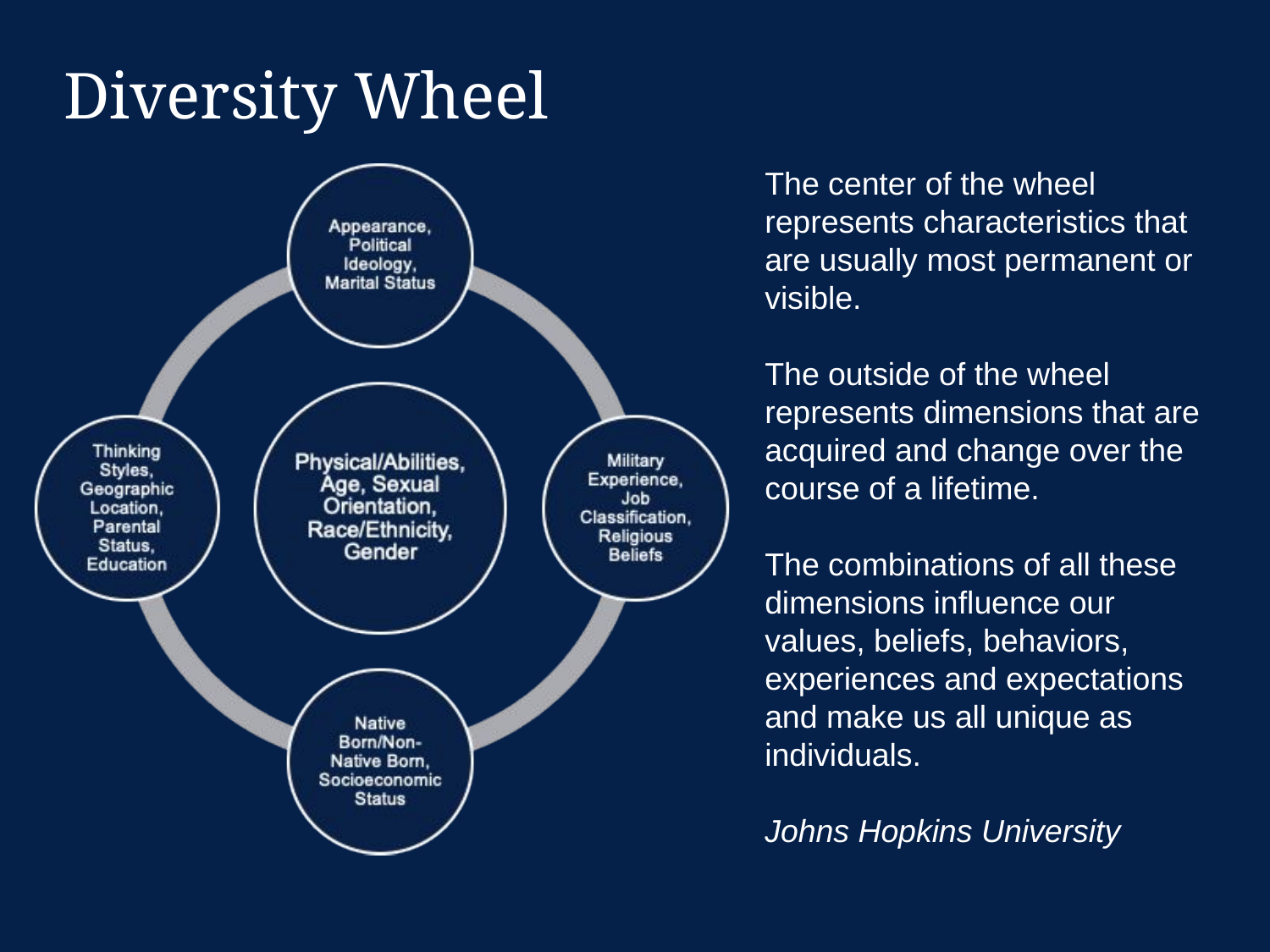

Diversity Wheel
The center of the wheel represents characteristics that are usually most permanent or visible.
The outside of the wheel represents dimensions that are acquired and change over the course of a lifetime.
The combinations of all these dimensions influence our values, beliefs, behaviors, experiences and expectations and make us all unique as individuals.
Johns Hopkins University

## Slide 18
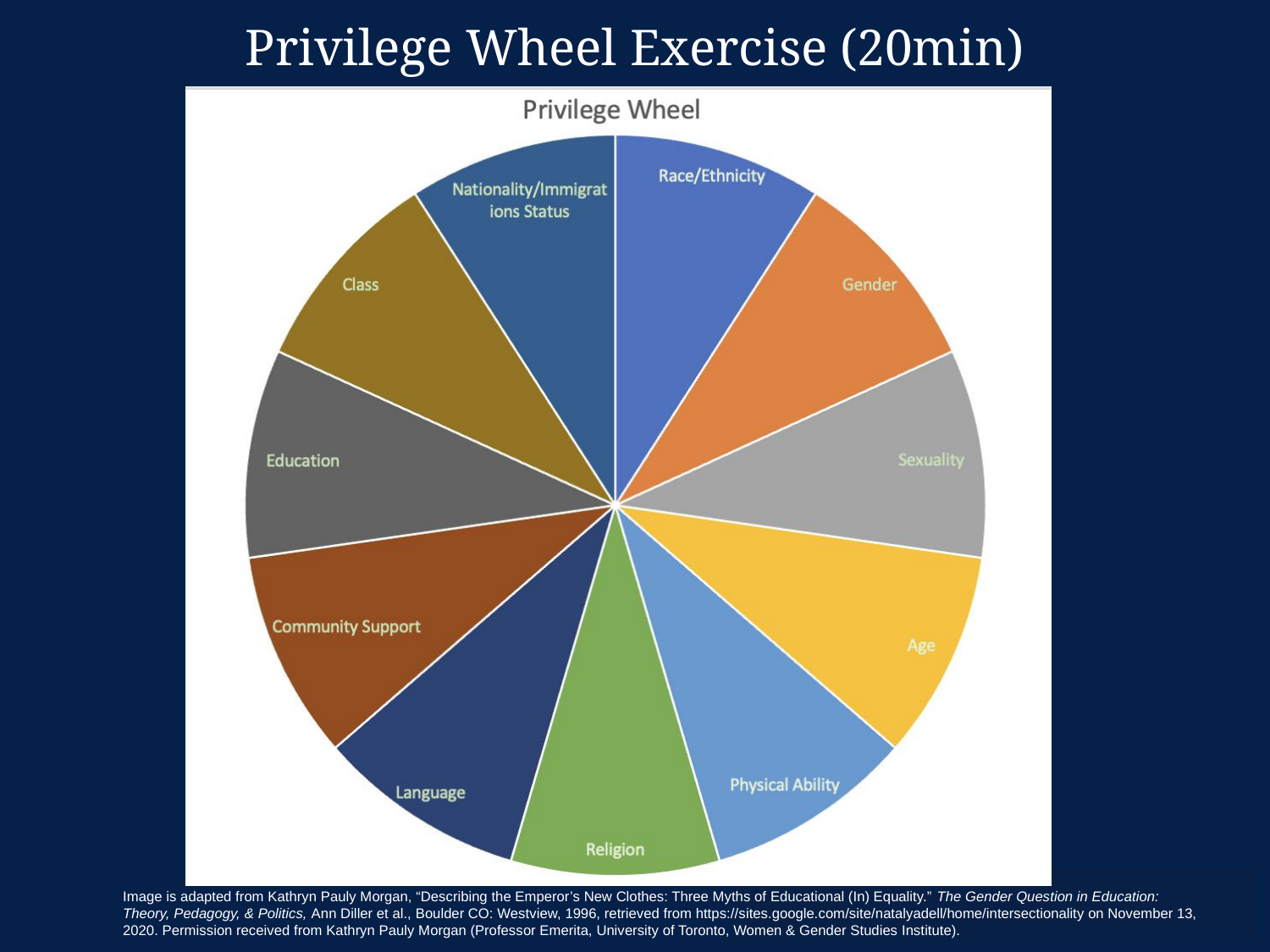

# Privilege Wheel Exercise (20min)
Image is adapted from Kathryn Pauly Morgan, “Describing the Emperor’s New Clothes: Three Myths of Educational (In) Equality.” The Gender Question in Education: Theory, Pedagogy, & Politics, Ann Diller et al., Boulder CO: Westview, 1996, retrieved from https://sites.google.com/site/natalyadell/home/intersectionality on November 13, 2020. Permission received from Kathryn Pauly Morgan (Professor Emerita, University of Toronto, Women & Gender Studies Institute).

## Slide 19
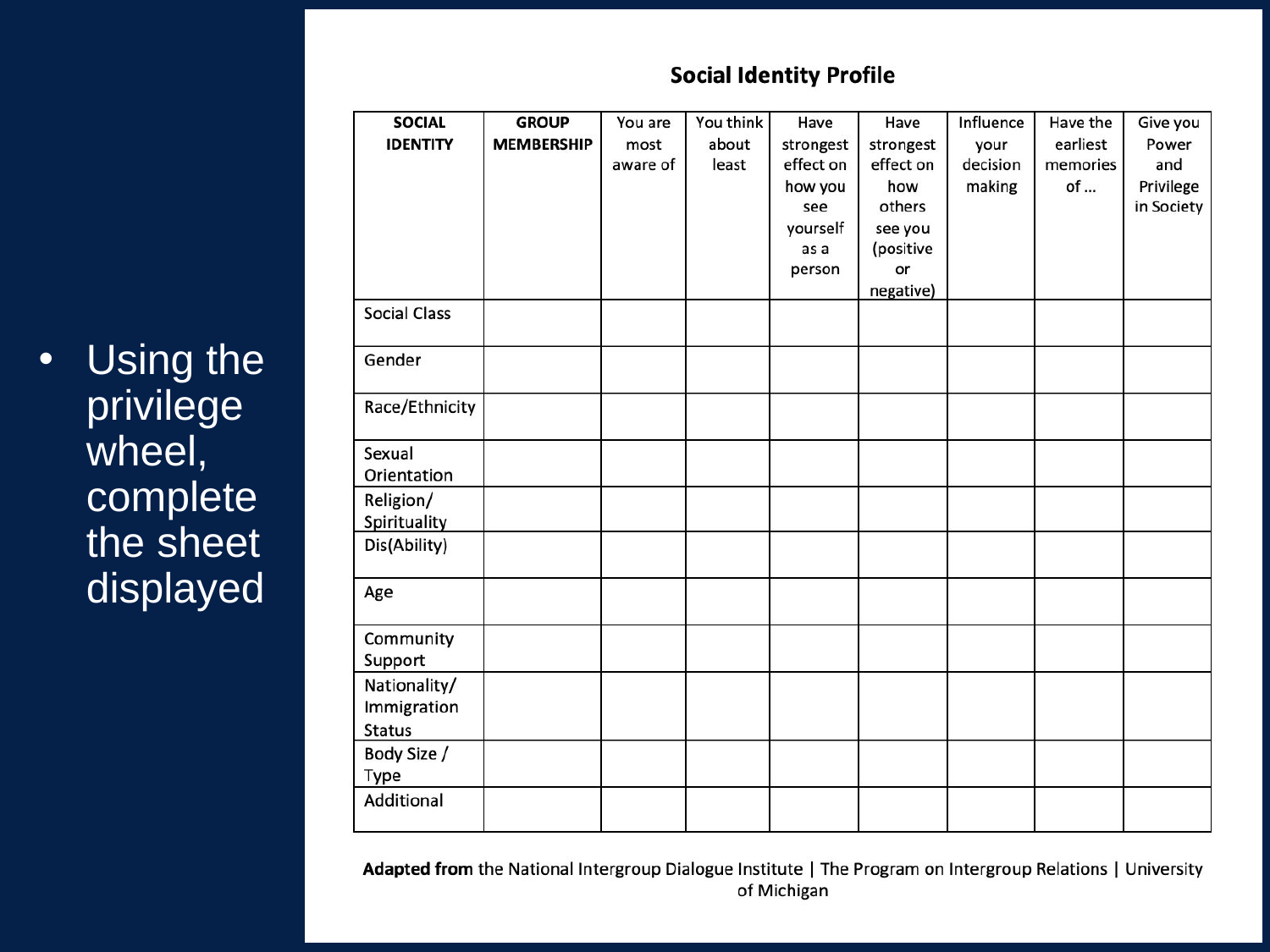

# Using the privilege wheel, complete the sheet displayed

## Slide 20
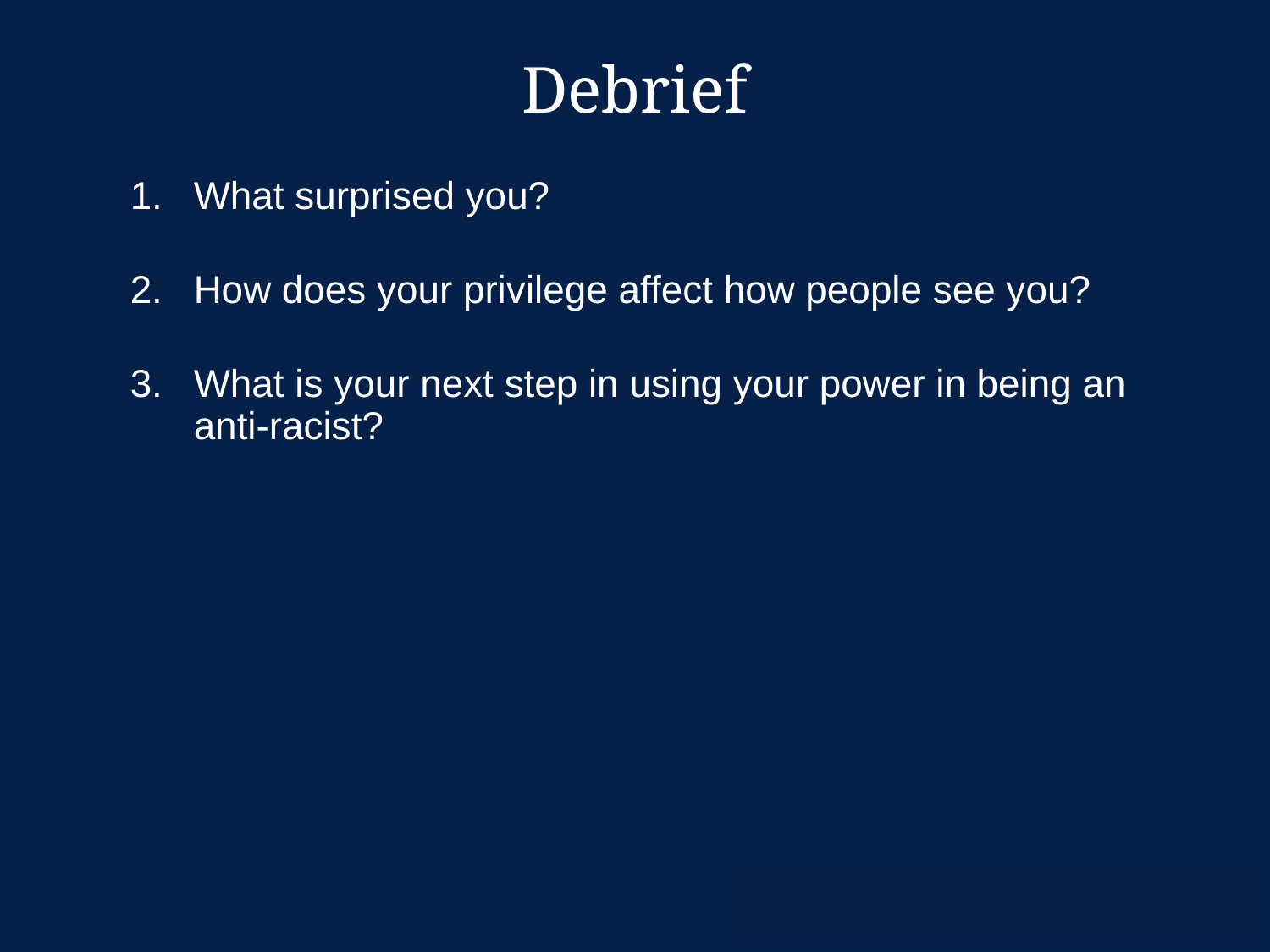

# Debrief
What surprised you?
How does your privilege affect how people see you?
What is your next step in using your power in being an anti-racist?

## Slide 21
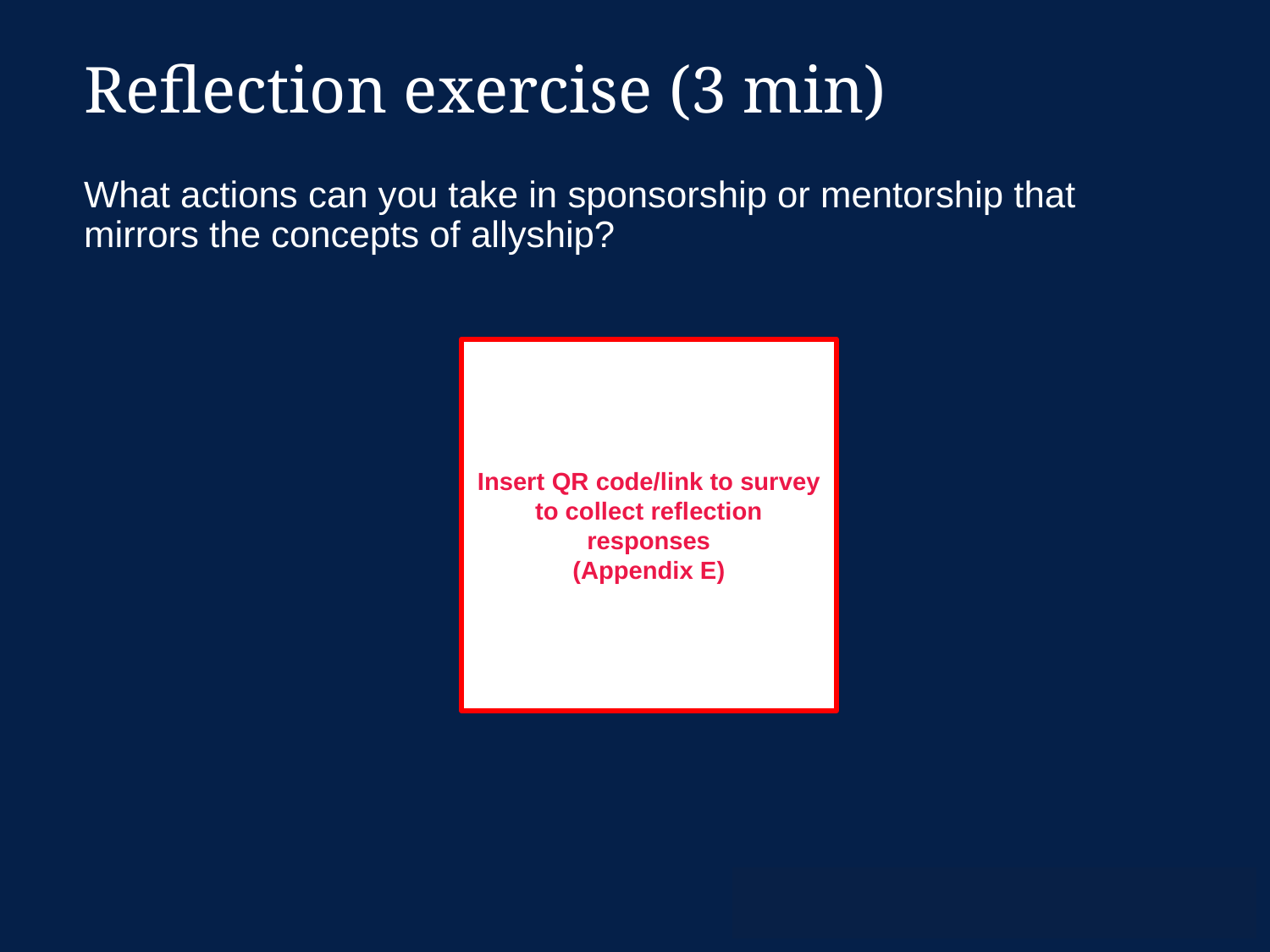

# Reflection exercise (3 min)
What actions can you take in sponsorship or mentorship that mirrors the concepts of allyship?
Insert QR code/link to survey to collect reflection responses
(Appendix E)
https://ucsf.co1.qualtrics.com/jfe/form/SV_7UKFz24AHWuZOnz

## Slide 22
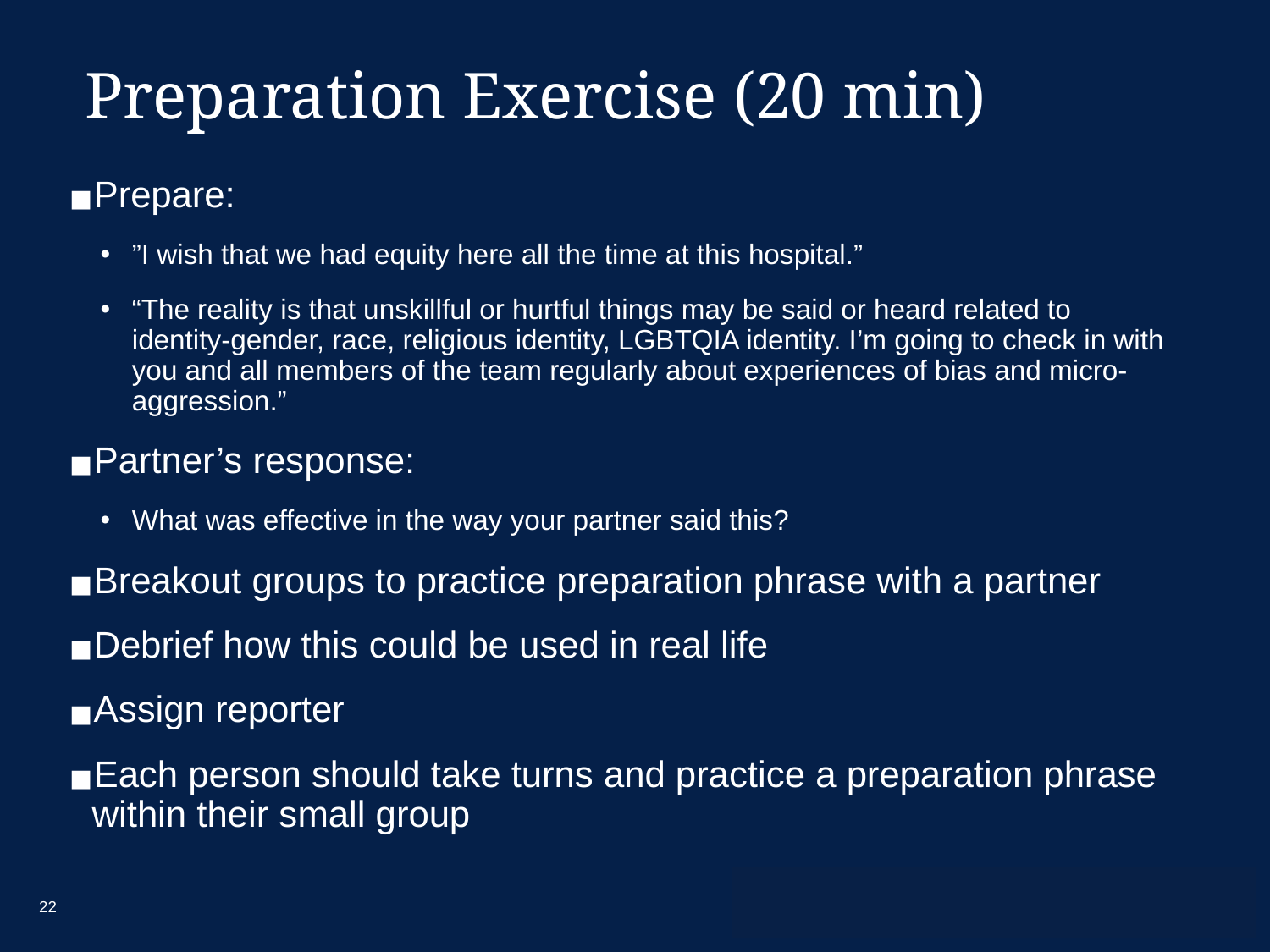

# Preparation Exercise (20 min)
Prepare:
”I wish that we had equity here all the time at this hospital.”
“The reality is that unskillful or hurtful things may be said or heard related to identity-gender, race, religious identity, LGBTQIA identity. I’m going to check in with you and all members of the team regularly about experiences of bias and micro-aggression.”
Partner’s response:
What was effective in the way your partner said this?
Breakout groups to practice preparation phrase with a partner
Debrief how this could be used in real life
Assign reporter
Each person should take turns and practice a preparation phrase within their small group
21

## Slide 23
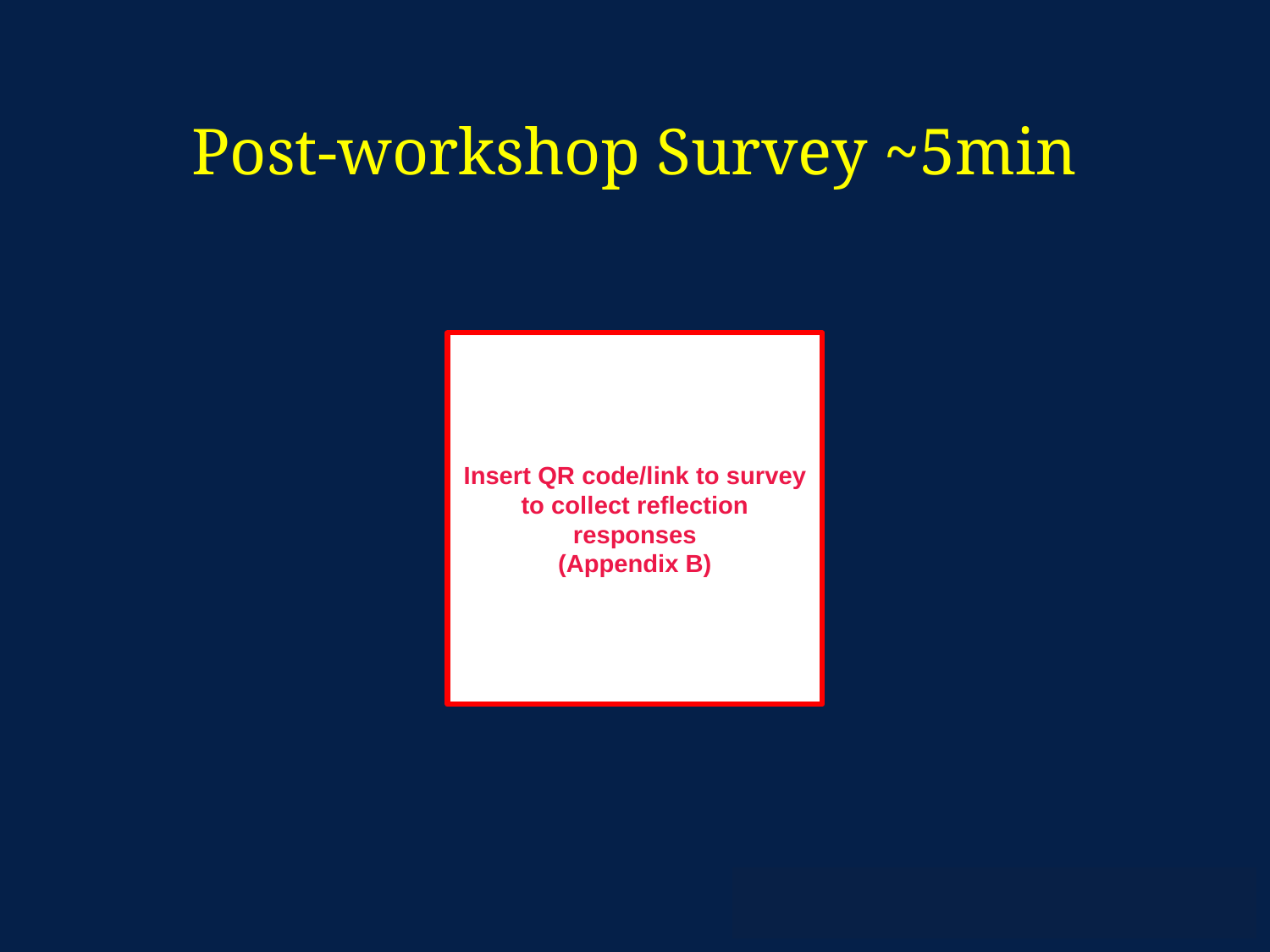

# Post-workshop Survey ~5min
Insert QR code/link to survey to collect reflection responses
(Appendix B)
https://ucsf.co1.qualtrics.com/jfe/form/SV_9Mq0LXNgscoTHgx

## Slide 24
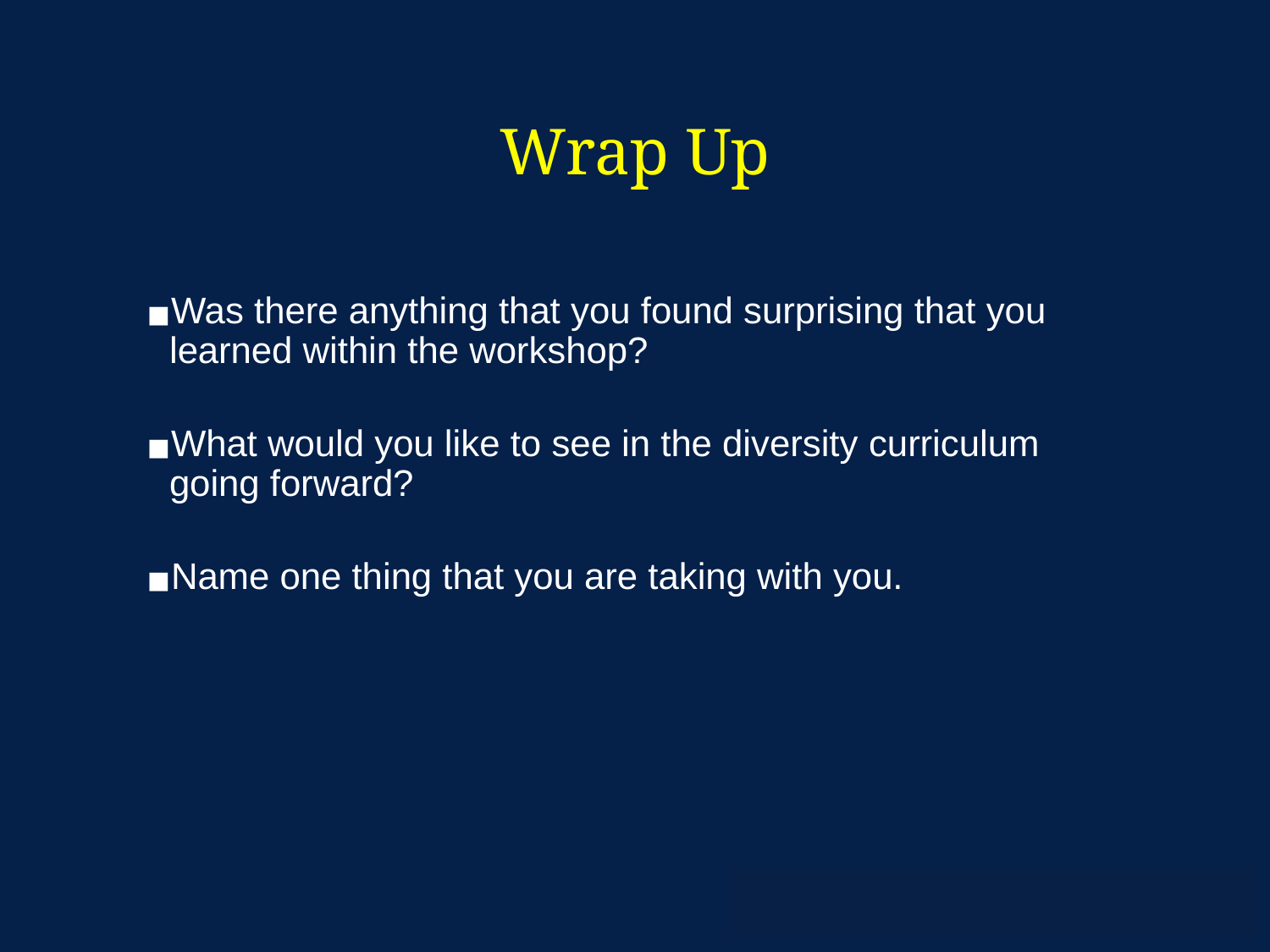

# Wrap Up
Was there anything that you found surprising that you learned within the workshop?
What would you like to see in the diversity curriculum going forward?
Name one thing that you are taking with you.

## Slide 25
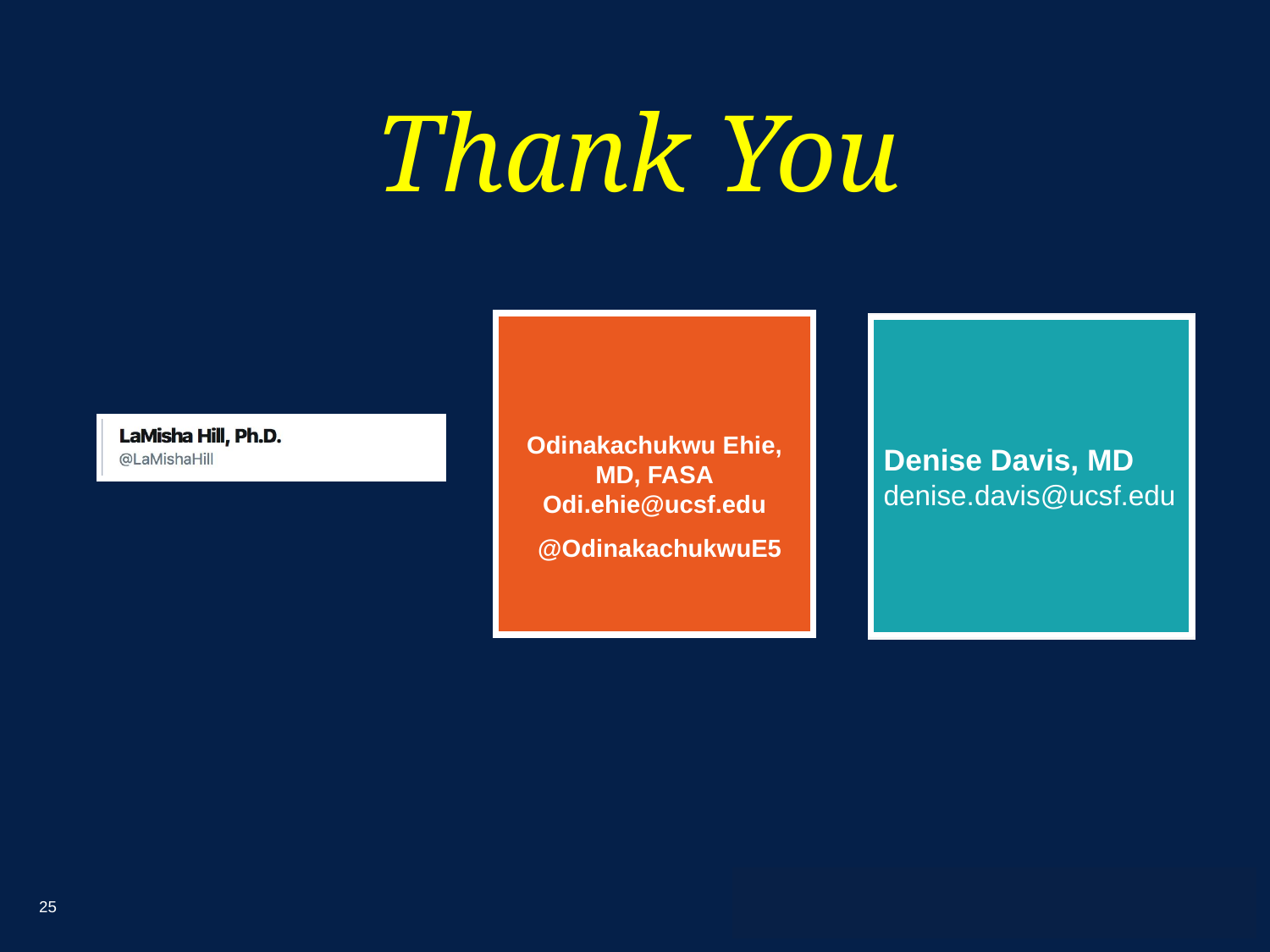

# Thank You
Odinakachukwu Ehie, MD, FASA
Odi.ehie@ucsf.edu
 @OdinakachukwuE5
Denise Davis, MD
denise.davis@ucsf.edu
24

## Slide 26
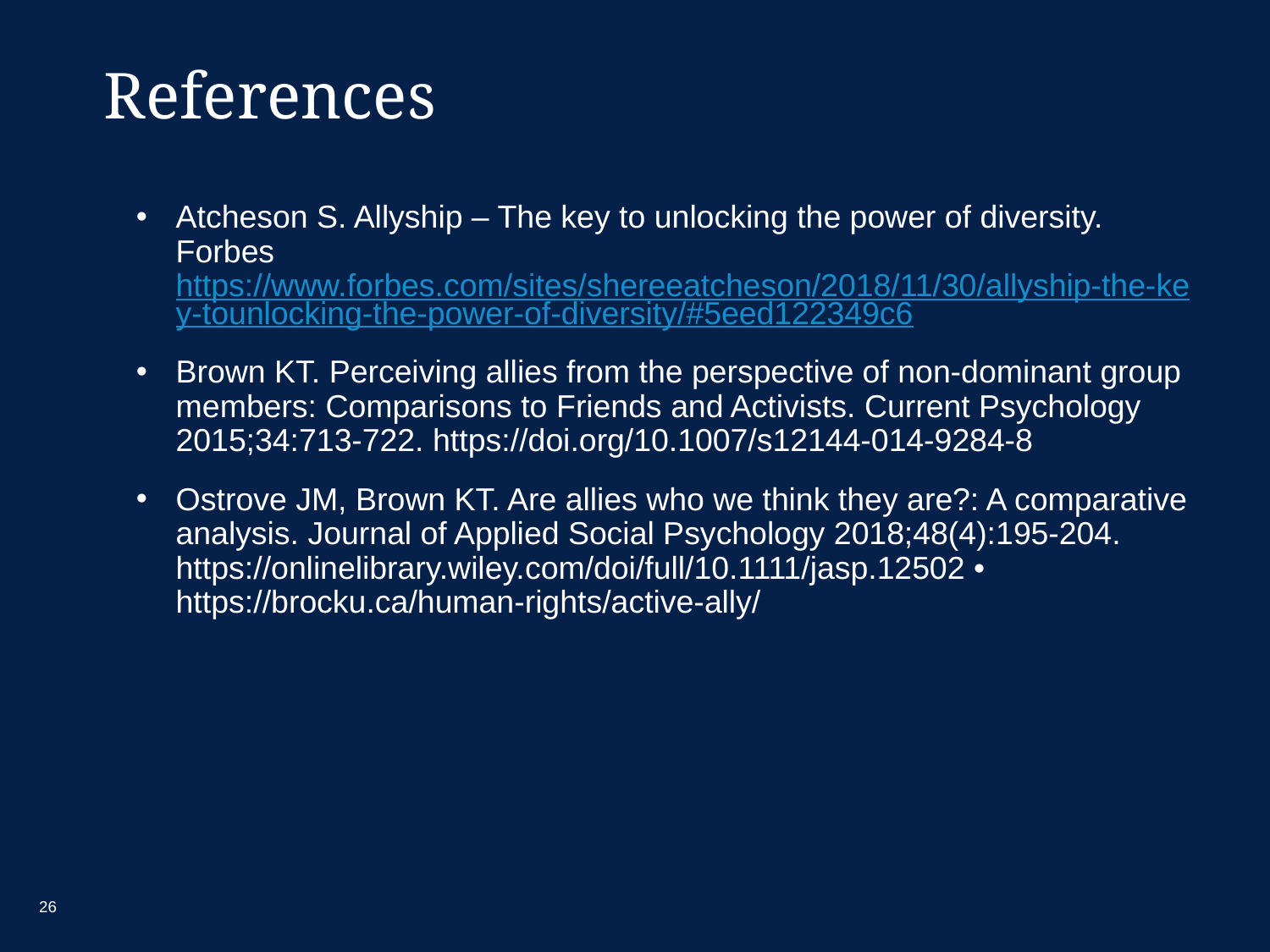

# References
Atcheson S. Allyship – The key to unlocking the power of diversity. Forbes https://www.forbes.com/sites/shereeatcheson/2018/11/30/allyship-the-key-tounlocking-the-power-of-diversity/#5eed122349c6
Brown KT. Perceiving allies from the perspective of non-dominant group members: Comparisons to Friends and Activists. Current Psychology 2015;34:713-722. https://doi.org/10.1007/s12144-014-9284-8
Ostrove JM, Brown KT. Are allies who we think they are?: A comparative analysis. Journal of Applied Social Psychology 2018;48(4):195-204. https://onlinelibrary.wiley.com/doi/full/10.1111/jasp.12502 • https://brocku.ca/human-rights/active-ally/
25
