# Supplementary material for: An Interactive Allyship and Privilege Workshop for Trainees in Medicine
Source: MedEdPORTAL. 2024 Aug 2;20:11426. doi: 10.15766/mep_2374-8265.11426 (PMC11294452; doi:10.15766/mep_2374-8265.11426)
Supplement: Supplementary file 1 — DEI Needs Assessment and Preworkshop Survey.docxFacilitator Guide.docxLearner Guide.docxAllyship Workshop Slides.pptxReflective Exercise.docxPostworkshop Survey.docx [file mep_2374-8265.11426-s001.zip › _Educational Summary Report_11426.pdf]

MedEdPORTAL® The AAMC Journal of Teaching and Learning Resources

Original Publication Open Access

An Interactive Allyship and Privilege Workshop for Trainees in Medicine

Janette Tang, MS, Rebecca Chen, LaMisha Hill Weller, PhD, Christy Boscardin, PhD, Odinakachukwu Ehie, MD*

*Corresponding author: odi.ehie@ucsf.edu

Abstract

Introduction: Despite growing efforts to increase diversity in recruitment and to teach principles of diversity, equity, and inclusion (DEI), representation of individuals underrepresented in medicine continues to fall short. This demonstrates a need for efforts that target the work environment and culture to increase retention alongside existing recruitment initiatives. We designed this interactive allyship workshop with a focus on building skills necessary for being an ally that has been missing in existing allyship curricula. **Methods:** This workshop was led by multidepartmental faculty with experience in DEI training. Participants engaged in a number of interactive activities to reflect on their own identities and privilege and practiced ways to engage in difficult conversations. Prior to the workshop, participants completed a survey that was repeated at the workshop's completion to evaluate their perspective change and understanding of allyship. We also collected responses to a self-reflective exercise during the workshop. **Results:** Participants included 68 anesthesia and surgery attendees, 53 of whom (78%) completed the postsurvey. Participants strongly agreed that this workshop was important to the workplace and medical training. Themes from the self-reflective exercises included endorsement of sponsorship and mentorship activities, community support, and advocacy. **Discussion:** Interactive skill-building activities are important and effective at helping trainees develop as allies. Long-term follow-up is needed to assess longitudinal knowledge retention and translation into behavioral change to create a more inclusive and supportive work environment.

Keywords
Allyship, Power, Privilege, Program Evaluation, Self-Assessment, Diversity, Equity, Inclusion

Educational Objectives

By the end of this activity, learners will be able to:

1. Explain two key examples of allyship that can be demonstrated in their training or specialty in the reflection exercise.
2. Distinguish two key differences between performative allyship and true allyship in a small-group activity.
3. Self-reflect on their own broad spectrum of privilege by identifying at least two privileges they hold in a small-group exercise.
4. Develop an action plan for sponsoring or mentoring that mirrors the concept of allyship in the reflection exercise.
5. Develop as allies and accomplices in equity by practicing conversations around discrimination through the role model exercise.

Citation:
Tang J, Chen R, Hill Weller L, Boscardin C, Ehie O. An interactive allyship and privilege workshop for trainees in medicine. *MedEdPORTAL*. 2024;20:11426.
https://doi.org/10.15766/mep_2374-8265.11426

Introduction

Initiatives for advancing diversity, equity, and inclusion (DEI) have exploded in recent years, with the increase of diversity in recruitment at the forefront of academic medicine.[1] While these efforts to advance diversity have yielded positive outcomes, gains in diversity have not been equitable across all groups, and much work remains in terms of addressing equity, inclusivity, and justice.[2] For example, in 2022-2023, women made up 57% of medical school matriculants.[3] However, the percentages of Black (10%) and Hispanic (12%) matriculants were still far from their census in the general population, which, according to the 2021 Census, was 13.6% Black or African American and 18.9% Hispanic or Latino.[3,4] Beyond matriculation, issues remain in the retention of trainees who identify as underrepresented in medicine (UIM) across the continuum of medical training.[5,6]

In medical school, UIM status correlates with increased attrition, and in residency, UIM trainees are more likely to withdraw, take extended leave, and be dismissed by their program.[6-8] The reasoning behind these trends is multifactorial—UIM trainees encounter obstacles ranging from navigating microaggressions and bias to being tasked as racial and ethnic ambassadors to

Copyright © 2024 Tang et al. This is an open-access publication distributed under the terms of the Creative Commons Attribution license.

social isolation.[9,10] These burdens compound the challenges of training and interplay with structural barriers (e.g., lack of mentorship and inequitable career advancement opportunities) to contribute to the dwindling number of racial and ethnic minority physicians at each successive stage of the academic pathway.[10-12]

Diversity in medicine also impacts health equity. Disparities in access to care remain an insurmountable hurdle in the care of underserved communities.[5] Studies have shown that UIM students and non-White physicians have a greater predilection towards working in underserved communities and that patients are more likely to seek racially concordant physicians.[13] As the populations of nonmajority groups continue to grow in the US, efforts that address diversity and retention in the health care workforce become increasingly important. Hence, allyship workshops that empower trainees to foster an inclusive environment and to practice sponsorship and mentorship are integral to creating an equitable health care system.[14-17]

Allyship is the continual process in which people with power and privilege work to develop a culture in which disadvantaged groups feel supported and valued.[18] There are few existing curricula that train medical residents to support their colleagues and patients specifically through the lens of allyship and privilege.[14,15,19] Most existing curricula primarily feature didactic models of teaching; few are activity based; none focus on skill building. One recent publication highlights the need for workshops aimed at clinical allyship skill building to better enable residents to develop as allies to support their peers and their patients.[19] In our own needs assessment survey (presurvey), participants expressed that they wanted to engage in small-group discussions to learn about actionable changes, uncover biases, and build skills to develop as allies.[20]

Based on these key observations, we created this allyship workshop as one part of a four-part DEI curriculum for surgery and anesthesia resident physicians at the University of California, San Francisco (UCSF).[20] This 2-hour virtual workshop can be used as a stand-alone session that involves large-group didactics and a number of small-group interactive, reflective, and skill-building exercises relevant to the medical professional. The workshop design references Kimberlé Crenshaw's critical race theory (CRT) and transformative learning to explore the concept of allyship and its deep roots in power and privilege.[21,22] The workshop can facilitate the process of becoming an ally by creating space for participants to explore and validate their own and their peers' identities and lived experiences. This active introspection on how those identities can affect the way one interacts with others models allyship. Training a health care workforce to support and advocate for patients and peers at an interpersonal level can culminate in broader positive cultural change across the institution.

Methods

Kern's six step approach to curriculum development was used in the creation of this innovative curriculum to teach perioperative residents about privilege and allyship.[23]

Needs Assessment (Presurvey)

We administered a general needs assessment survey (Appendix A) 6-8 weeks prior to the allyship workshop during the DEI curriculum introductory session for first-year anesthesia residents that preceded this curriculum in 2020 and 2021.[20] This survey was validated through a pilot with medical students and residents for clarity and relevance.[20] We also emailed the survey to all general surgery and anesthesia residents at UCSF to reach a wider audience. The survey captured participant demographics, as well as what the residents felt was lacking in their education regarding DEI training, and served as a presurvey for the allyship workshop, including knowledge and perception questions about allyship and privilege.

Facilitator Preparation

Experienced facilitators were selected across various specialties representing diverse lived experiences and backgrounds. To be experienced meant having taught a similar type of workshop in the past or having participated in multiple DEI trainings. One week before the workshop, facilitators attended a mandatory 1-hour train-the-trainer session introducing the workshop activities and the facilitator guide (Appendix B). Facilitators also received guidance on how to approach difficult topics and conversations that might arise.

Learning Environment

To prepare for the workshop, participants were provided with a learner guide with course objectives and recommended reading (Appendix C). Due to COVID-19, the session was administered through a virtual video meeting platform (Zoom). We set a maximum facilitator to learner ratio of 1:5 to promote vulnerable conversation.

We began the workshop with a brief house agreement to set house rules and expectations for learning and unlearning. Participants and facilitators agreed upon the following: (1) Assume positive intent, (2) listen actively, (3) participate fully and bravely by leaving your comfort zone and entering the

Copyright © 2024 Tang et al. This is an open-access publication distributed under the terms of the Creative Commons Attribution license.

growth zone, (4) step up and step back, (5) share your story using "I" statements, and (6) confidentiality. We encouraged participants to keep their videos on in order to maximize interpersonal interaction.

Participants
This workshop was not designed specifically for perioperative trainees. However, the umbrella DEI curriculum originated from the UCSF Department of Anesthesia, so the learners in the workshop incidentally included only perioperative residents. We worked with the respective residency administrators to secure protected didactic time for all anesthesia CA 1 residents and surgery PGY 4 and PGY 5 residents.

Educational Content
Selection of the educational material was guided by the needs assessment; the material was delivered in a combination of didactics and small-group activities. In the process of creating and curating the educational content, we referenced CRT and intersectionality as frameworks to guide conversations in the workshop and to emphasize the importance of how each person's identities can impact their lived experiences.[21] We aimed to create a space for participants to engage in these important conversations to make way for transformative learning and development as true allies (Figure 1). The didactics fetaured important vocabulary for engaging in conversations around allyship, including such terms as true allyship, performative allyship, privilege, and power, as well as important topics like what it meant to be an ally, ways allyship could manifest, and the difference between sponsors and mentors (Appendix D).

The interactive activities included Circles of My Multicultural Self, the Privilege Wheel, a preparation exercise, and a reflection exercise.[24,25] The Circles of My Multicultural Self and Privilege Wheel activities were adapted from use in other disciplines to accomplish our workshop objectives.[15] The preparation and reflection exercises were novel activities. Each small group had a facilitator and five to six participants who stayed together throughout the workshop. After each small-group activity, participants returned to the large group to debrief. A reporter from each small group was self-selected to summarize their respective discussions directed by the exercises in the facilitator guide (Appendix B).

The Circles of My Multicultural Self activity highlighted the various identities that different individuals might hold and challenged the stereotypes that could be associated with the ways people distinguish themselves. The adapted Privilege Wheel exercise aimed to illuminate the diversity of shared and unique identities present and to foster empathy by learning about one another's lived experiences.[15] The reflection exercise was a real-time anonymous Qualtrics survey with questions challenginig the participant to identify actions they could practice that would demonstrate allyship (Appendix E). The preparation exercise was a novel skill-building activity created by one of our facilitators in which participants observed and practiced an effective strategy for approaching the topic of inequity and discrimination in a small-group setting.

Postsurvey Assessment and Evaluation
We used the Kirkpatrick model as the framework for curriculum evaluation and a mixed-methods analysis to capture both the impact of the workshop and self-reflective exercises around privilege and allyship.[26,27] The postsurvey included both 5-point Likert-scale and open-ended items to collect demographics, assess learners' satisfaction with the workshop and facilitators, and evaluate perception change and participants' understanding around the concepts of allyship.[28] The postsurvey (Appendix F) was administered to participants in the last 5 minutes of the workshop. Participants also submitted anonymous self-reflections, as stated above (Appendix E). Responses to both surveys and the reflective exercises were collected using Qualtrics.

Data Analysis
Quantitative analysis included *t* tests of the pre- and postsurveys using SPSS (IBM). Anonymous identifiers were self-generated by the participants for matching in pre/post analysis. Qualitative data analysis included a content analysis research methodology with consensus coding to analyze the data. Two researchers performed initial coding separately based on frequency of a concept or implied concept using Microsoft Excel.[29] They next came together to identify overlapping codes and share reasoning for coding inconsistencies in order to eventually reach consensus. One of the researchers then revisited the final codes to draw conclusions and findings based on the codes that had been identified.

The UCSF Institutional Review Board deemed this study exempt from review on May 14, 2020 (IRB approval no. 19-29554).

Results

Participant Demographics
This workshop was administered to 44 anesthesia CA 1 residents and 24 surgery PGY 4/PGY 5 residents over the course of four separate sessions in October 2020 and October 2021. A total of 55 residents—39 out of 44 anesthesia CA 1s (89%) and

Copyright © 2024 Tang et al. This is an open-access publication distributed under the terms of the Creative Commons Attribution license.

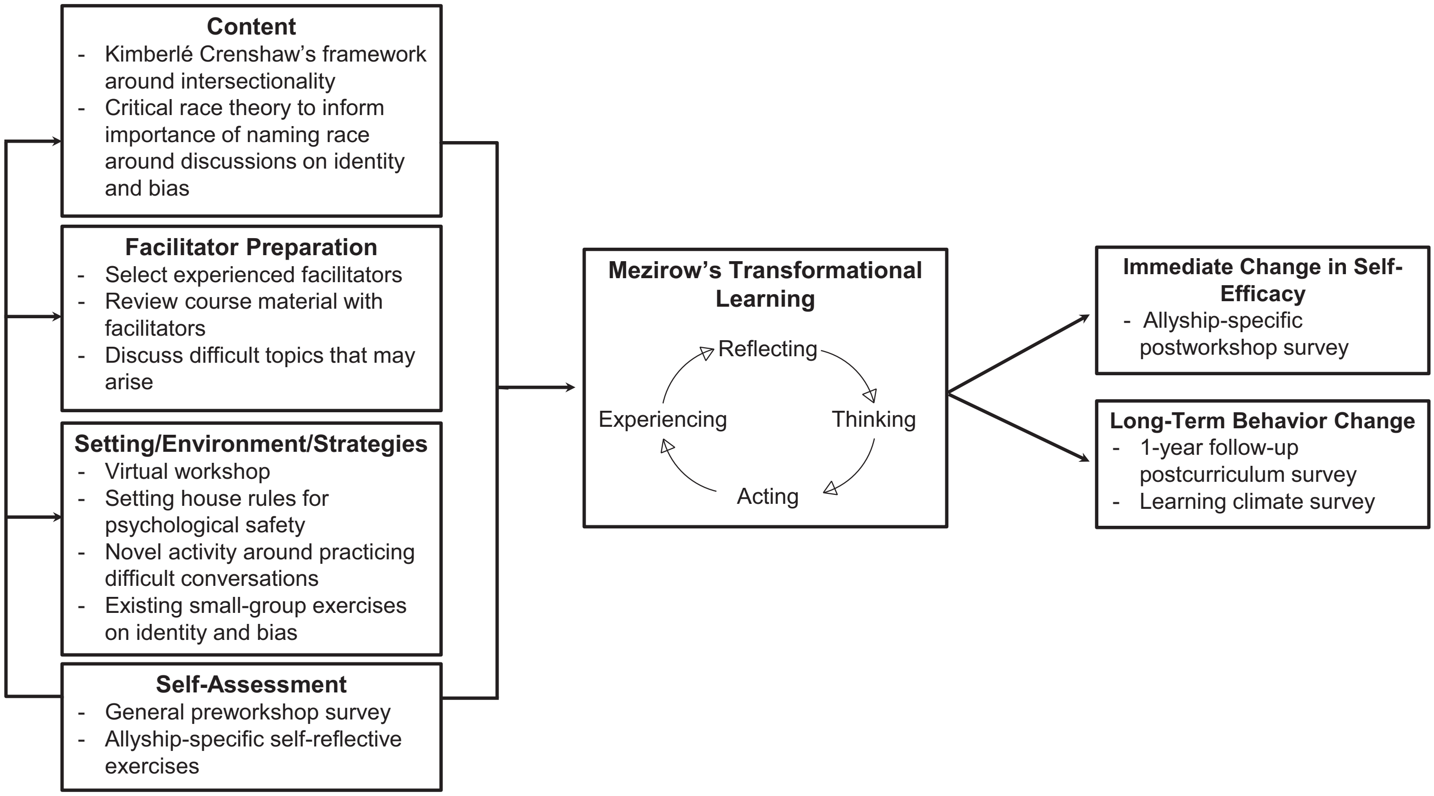

Figure 1. The transformational learning framework. This conceptual framework shows that transformative learning in the allyship workshop is an ongoing process facilitated by knowledge acquisition, well-prepared facilitators, interactive activities and skill building, and self-reflection, with the ultimate goal of long-term behavioral change.

13 out of 24 surgery PGY 4s (54%)—were represented in the postsurvey responses. Among the anesthesia participants, 21 (54%) identified as female, seven (18%) identified as LGBTQIA+, and 16 (41%) identified as UIM. Among the surgery participants, five (38%) identified as female, one (8%) identified as LGBTQIA+, and three (23%) identified as UIM.

Workshop and Facilitator Satisfaction

Postsurvey responses regarding satisfaction with the workshop and nine facilitators were overwhelmingly positive. On the 5-point Likert-scale (1 = *strongly disagree,* 5 = *strongly agree*), the 55 participants evaluated the statements "This allyship workshop is important to my training" with a mean of 4.6 ($SD = 0.8$), "I believe this allyship workshop is relevant to my workplace" with a mean of 4.7 ($SD = 0.8$), and "I would recommend this allyship workshop to my peers" with a mean of 4.6 ($SD = 0.8$; Table 1).

Regarding facilitator satisfaction, participants agreed that the facilitators "were well prepared" with a mean of 4.8 ($SD = 0.6$), "created a welcoming and inclusive environment for discussions" with a mean of 4.8 ($SD = 0.6$), and "effectively communicated this information" with a mean of 4.8 ($SD = 0.6$; Table 1).

Self-Perceived Competence

Self-competence was assessed through three statements in the 2021 workshops, which consisted of 30 participants. The mean response to the statement "I know how to define the term allyship" increased from 3.7 ($SD = 1.1$) to 4.5 ($SD = 0.5$) from the pre- to postsurvey ($p < .05$). The mean response to "As an ally, I am likely to mentor individuals who belong to a marginalized group and recommend them for other academic projects" increased from 3.8 ($SD = 1.1$) to 4.5 ($SD = 0.6$) from the pre- to postsurvey ($p < .05$). There was no significant change in the mean response to "I recognize the privilege that I have" from the pre- to postsurvey (Figure 2).

Self-Reflective Exercise

Twenty-five participants submitted self-reflections on the question "What actions can we take in sponsoring or mentoring that mirror the concept of allyship?", summarized by the following themes in the order of prominence: advocating for others (44%), actively seeking out mentorship and sponsorship opportunities (40%), fostering a supportive community (32%), respecting others' identities and experiences (16%), positive role modeling (12%), and acknowledging and leveraging one's own privilege (8%). The

Copyright © 2024 Tang et al. This is an open-access publication distributed under the terms of the Creative Commons Attribution license.

Table 1. Workshop and Facilitator Feedback (N = 55)

Statement	Strongly Disagree	Somewhat Disagree	Neutral	Somewhat Agree	Strongly Agree
This allyship workshop is important to my training.	2%	2%	4%	16%	76%
I believe this allyship workshop is relevant to my workplace.	2%	2%	0%	20%	76%
I would recommend this allyship workshop to my peers.	2%	2%	2%	26%	68%
The facilitators were well prepared.	2%	0%	0%	13%	85%
The facilitators created a welcoming and inclusive environment for discussions.	2%	0%	0%	11%	87%
The facilitators effectively communicated this information.	2%	0%	0%	11%	87%

12 responses to the question "What did you learn about yourself [through this workshop]?" included the intersectionality of one's own identities (75%) and that one was privileged in ways one did not realize (50%). Moving forward, participants (14) stated that they would become more self-aware of their privilege (50%), be an ally (43%), speak up and speak out (29%), and practice active listening (21%; Table 2).

Workshop Feedback

At the end of the postsurvey, participants were asked to provide feedback about the workshop. The 31 responses to the question "What did you like most about this workshop?" can be summarized by the following themes: small-group exercises (42%), safe space for open conversation and discussion (39%), effective and well-prepared facilitators (16%), reflecting on one's own identities and privilege (16%), learning from those with different backgrounds (16%), and specific actions and ways to be an ally (10%). The 20 responses to the question "What could be improved in this workshop?" included more time in small groups (55%), less lecture time (20%), encouraging more participation (15%), and more tips for being an ally (10%; Table 2).

Discussion

We developed this novel interactive allyship workshop to create space for participants to self-reflect on their identities and privilege and to facilitate the skill building necessary to help trainees develop as allies. The postsurvey results were overwhelmingly positive. The high satisfaction rate with facilitators indicated their effectiveness despite the limitations of the virtual format due to the COVID-19 pandemic. The results demonstrated that the exercises implemented were useful in encouraging participants to discuss and reflect on their own and their peers' identities. The self-reflection activity also received a unanimously positive response indicating high motivation

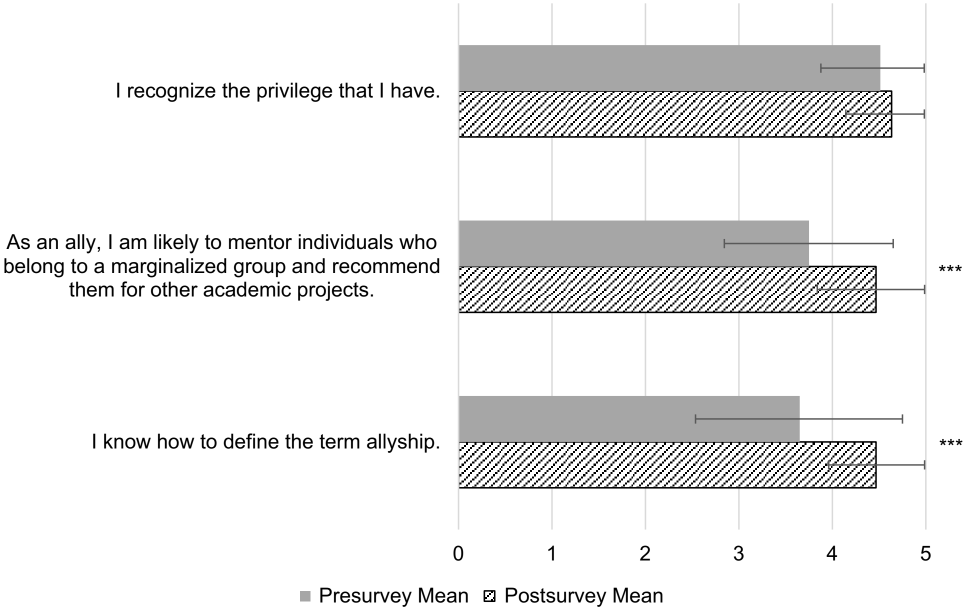

Figure 2. Change in self-competence. Thirty participants rated three statements on a 5-point Likert scale (1 = *strongly disagree,* 5 = *strongly agree*). The figure shows mean pre/post change in self-perceived competence. Error bars indicate one standard deviation. Asterisks (***) indicate statistically significant change from pre- to postworkshop survey responses ($p < .05$).

Copyright © 2024 Tang et al. This is an open-access publication distributed under the terms of the Creative Commons Attribution license.

Table 2. Themes Derived From Reflective Exercise and Workshop Feedback

Question	Themes
What actions can we take in sponsoring or mentoring that mirror the concept of allyship? (*N* = 25)	Advocate for others (44%)
	Actively seek out mentorship and sponsorship opportunities (40%)
	Foster a supportive community (32%)
	Respect others' identities and experiences (16%)
	Positive role modeling (12%)
	Acknowledge and leverage my own privilege (8%)
What did you learn about yourself? (*N* = 12)	The intersectionality of my identities (75%)
	I am privileged in ways I did not realize (50%)
What will you do differently moving forward? (*N* = 14)	Become more self-aware of my privilege (50%)
	Be an ally (43%)
	Speak up and speak out (29%)
	Practice active listening (21%)
What did you like most about this workshop? (*N* = 31)[a]	Small-group exercises (42%)
	Safe space for open conversation and discussion (39%)
	Effective and well-prepared facilitators (16%)
	Reflecting on my own identities and privilege (16%)
	Learning from those with different backgrounds (16%)
	Specific actions and ways to be an ally (10%)
What could be improved in this workshop? (*N* = 20)[a]	More time in small-group/breakout sessions (55%)
	Less lecture time (20%)
	Encourage more participation (15%)
	More tips for being an ally (10%)

[a]Workshop feedback question.

among participants to apply the allyship training to create a more supportive and inclusive community. The critical thinking and self-reflection employed throughout the workshop represent key steps towards developing as allies within Mezirow's transformational learning framework (Figure 1).[30] Our positive findings could help bridge our long-term goal of behavioral change to building a health care workforce of allies who uplift their disadvantaged colleagues and peers.

Through the curriculum feedback, we learned important lessons for future iterations of the workshop. We intend to limit the amount of didactics and allot more time for working together in the intimate, brave spaces of our smaller breakout groups, which focus on engaging in the exercises and discussions. Next, the implementation of an allyship workshop can be challenging due to the difficulty of creating a vulnerable and brave space that empowers participants to share their thoughts and perspectives. We found that facilitators who opened up and shared their own vulnerable experiences first set the tone for the type of discussion that the group would have. As a result, future facilitators will be encouraged to be vulnerable with their learners to help build space for learning and growth.

We delivered the workshop in a virtual format due to the COVID-19 pandemic, which posed another challenge to creating a vulnerable environment in which to learn compared to an in-person workshop. We were concerned that the workshop's interactivity would be heavily impacted by the virtual format but instead discovered that learners were engaged and ready to participate. Experienced facilitators and effective preparatory session materials were likely strong contributors to the highly interactive experience. Furthermore, the virtual format permitted greater flexibility to recruit a more diverse, multidisciplinary group of facilitators and to manage participants' time and location constraints. One could still administer this workshop in an in-person format. We had initially intended to deliver it in person prior to the pandemic. The main adjustment for the virtual format was the emphasis on encouraging participants to keep their videos on during the house rules.

The workshop has several limitations. It is not longitudinal, and thus, our short-term assessments are not reliable measurements of the long-term change we want to achieve (Figure 1). We intend to administer a climate survey as a long-term follow-up to assess the longitudinal retention of allyship knowledge and its transition into behavioral change. Furthermore, technical challenges in the survey launch resulted in the omission of questions assessing self-competency from the 2020 postsurvey; this was corrected in the 2021 survey. Despite the survey having been piloted, several questions remained unclear when survey was actually implemented; we therefore omitted these questions from the final results. Survey responses were voluntary, and so, certain items on the survey received a smaller number of responses than others despite the overall high response rate. There

Copyright © 2024 Tang et al. This is an open-access publication distributed under the terms of the Creative Commons Attribution license.

were also significantly more anesthesia participants relative to surgery residents, which highlights the importance of having departmental support to navigate challenging trainee schedules.

To foster a more inclusive work environment in academic medicine, a greater emphasis on retention of a diverse workforce must take priority. Our workshop directly addresses this prevalent issue by creating space for participants to self-reflect and build the skills necessary to develop as allies. The workshop also empowers learners to become advocates as mentors and sponsors, which can help pave a road to increasing marginalized representation through recruitment and retention at all levels of training. We recommend continued evolution and dissemination of this workshop so that allyship training becomes standard within academic medicine to promote institutional change that truly emphasizes a culture of DEI and justice.

Appendices

A. DEI Needs Assessment and Preworkshop Survey.docx

B. Facilitator Guide.docx

C. Learner Guide.docx

D. Allyship Workshop Slides.pptx

E. Reflective Exercise.docx

F. Postworkshop Survey.docx

All appendices are peer reviewed as integral parts of the Original Publication.

Janette Tang, MS: Fourth-Year Medical Student, University of California, San Francisco, School of Medicine

Rebecca Chen: Fourth-Year Medical Student, University of California, San Francisco, School of Medicine

LaMisha Hill Weller, PhD: Adjunct Associate Professor, Department of Obstetrics and Gynecology, University of California, San Francisco, School of Medicine

Christy Boscardin, PhD: Professor, Department of Medicine and Anesthesia, University of California, San Francisco, School of Medicine

Odinakachukwu Ehie, MD: Associate Professor, Department of Anesthesia and Perioperative Care, University of California, San Francisco, School of Medicine; ORCID: https://orcid.org/0000-0001-8436-8897

Acknowledgments
We would like to acknowledge Gillian Earnest for her statistical analysis support in this publication.

Disclosures
None to report.

Funding/Support
Odinakachukwu Ehie received research funding from the Research in Funding Grant—Foundation of Anesthesia Education and Research (FAER) as the principal investigator of the diversity, equity, and inclusion curriculum for anesthesia and surgery residents.

Janette Tang and Rebecca Chen received research funding from the University of California, San Francisco, School of Medicine Summer Explore Research Fellowship.

Ethical Approval
The University of California, San Francisco, Institutional Review Board deemed further review of this project not necessary.

References

1. Awosogba T, Betancourt JR, Conyers FG, et al. Prioritizing health disparities in medical education to improve care. *Ann N Y Acad Sci*. 2013;1287(1):17-30. https://doi.org/10.1111/nyas.12117
2. *Diversity in Medicine: Facts and Figures 2019*. Association of American Medical Colleges; 2019. Accessed July 1, 2024. https://www.aamc.org/media/38266/download?attachment
3. Diversity increases at medical schools in 2022. Association of American Medical Colleges. December 13, 2022. Accessed July 1, 2024. https://www.aamc.org/news/press-releases/diversity-increases-medical-schools-2022#:~:text=Diversity%20of%20enrollees,9.5%25%20in%202020%2D21
4. Population estimates, July 1, 2023. United States Census Bureau. Accessed July 1, 2024. https://www.census.gov/quickfacts/fact/table/US#
5. McDade WA. Increasing graduate medical education diversity and inclusion. *J Grad Med Educ*. 2019;11(6):736-738. https://doi.org/10.4300/JGME-D-19-00760.1
6. McDade W. *ACGME's Approach to Diversity, Equity, and Inclusion in GME*. Accreditation Council for Graduate Education; 2022. Accessed July 1, 2024. https://healthinfo.montana.edu/events-training/3.McDade%20WWAMI%20DEI%20Panel%202022.pdf
7. Harris R, Covington K, Colford C, et al. Focusing on diversity: a regional internal medicine residency viewpoint on underrepresented minority support, retention, and mentoring. *J Grad Med Educ*. 2021;13(2):181-188. https://doi.org/10.4300/JGME-D-20-00729.1
8. Nguyen M, Chaudhry SI, Desai MM, et al. Association of sociodemographic characteristics with US medical student attrition. *JAMA Intern Med*. 2022;182(9):917-924. https://doi.org/10.1001/jamainternmed.2022.2194
9. Osseo-Asare A, Balasuriya L, Huot SJ, et al. Minority resident physicians' views on the role of race/ethnicity in their training experiences in the workplace. *JAMA Netw Open*. 2018;1(5):

Copyright © 2024 Tang et al. This is an open-access publication distributed under the terms of the Creative Commons Attribution license.

e182723.
https://doi.org/10.1001/jamanetworkopen.2018.2723

10. Argueza BR, Saenz SR, McBride D. From diversity and inclusion to antiracism in medical training institutions. *Acad Med*. 2021;96(6):798-801. https://doi.org/10.1097/ACM.0000000000004017
11. Campbell KM, Hudson BD, Tumin D. Releasing the net to promote minority faculty success in academic medicine. *J Racial Ethn Health Disparities*. 2020;7(2):202-206. https://doi.org/10.1007/s40615-020-00703-z
12. Blackstock U. Why Black doctors like me are leaving faculty positions in academic medical centers. STAT. January 16, 2020. Accessed July 1, 2024. https://www.statnews.com/2020/01/16/black-doctors-leaving-faculty-positions-academic-medical-centers
13. Marrast LM, Zallman L, Woolhandler S, Bor DH, McCormick D. Minority physicians' role in the care of underserved patients: diversifying the physician workforce may be key in addressing health disparities. *JAMA Intern Med*. 2014;174(2):289-291. https://doi.org/10.1001/jamainternmed.2013.12756
14. Djulus G, Sheikhan NY, Nawaz E, et al. Advancing allyship through anti-oppression workshops for public health students: a mixed methods pilot evaluation. *Pedagogy Health Promot*. 2021;7(4):304-312. https://doi.org/10.1177/2373379920962410
15. Chow CJ, Case GA, Matias CE. Tools for discussing identity and privilege among medical students, trainees, and faculty. *MedEdPORTAL*. 2019;15:10864. https://doi.org/10.15766/mep_2374-8265.10864
16. Garran AM, Aymer S, Gelman CR, Miller JL. Team-teaching anti-oppression with diverse faculty: challenges and opportunities. *Soc Work Educ (Lond)*. 2015;34(7):799-814. https://doi.org/10.1080/02615479.2015.1062086
17. Monrouxe LV. Identity, identification and medical education: why should we care? *Med Educ*. 2010;44(1):40-49. https://doi.org/10.1111/j.1365-2923.2009.03440.x
18. Atcheson S. Allyship—the key to unlocking the power of diversity. Forbes. 2018. Accessed July 1, 2024. https://www.forbes.com/sites/shereeatcheson/2018/11/30/allyship-the-key-to-unlocking-the-power-of-diversity/?sh=5f37e3e49c6f
19. Martinez S, Araj J, Reid S, et al. Allyship in residency: an introductory module on medical allyship for graduate medical trainees. *MedEdPORTAL*. 2021;17:11200. https://doi.org/10.15766/mep_2374-8265.11200
20. Ehie O, Tang J, Chen R, Turnbull J, Hill L. Implementation of a pilot diversity curriculum to anesthesia trainees. *J Clin Res Pain Anaesth*. 2021;3(1):180024.
21. Delgado R, Stefancic J. *Critical Race Theory: An Introduction*. 3rd ed. New York University Press; 2017.
22. Kaufman DM. Applying educational theory in practice. *BMJ*. 2003;326(7382):213-216. https://doi.org/10.1136/bmj.326.7382.213
23. Thomas PA, Kern DE, Hughes MT, Tackett SA, Chen BY, eds. *Curriculum Development for Medical Education: A Six-Step Approach*. 4th ed. Johns Hopkins University Press; 2022.
24. *Diversity Activities Resource Guide*. University of Houston; 2019. Accessed June 15, 2020. https://www.uh.edu/cdi/resources/student-resources/_files/_activities/diversity-activities-resource-guide.pdf
25. Diller A, Houston B, Morgan KP, Ayim M. *The Gender Question in Education: Theory, Pedagogy, and Politics*. Routledge; 2018.
26. Kirkpatrick DL. Techniques for evaluation training programs. *J Am Soc Train Dir.* 1959;13:21-26.
27. Hu YY, Ellis RJ, Hewitt DB, et al. Discrimination, abuse, harassment, and burnout in surgical residency training. *New Engl J Med*. 2019;381(18):1741-1752. https://doi.org/10.1056/NEJMsa1903759
28. Creswell JW, Plano Clark VL. *Designing and Conducting Mixed Methods Research*. 3rd ed. Sage; 2018.
29. Green J, Thorogood N. *Qualitative Methods for Health Research*. Sage; 2004:173-200.
30. Mezirow J. Transformative learning: theory to practice. *New Dir Adult Contin Educ*. 1997;(74):5-12. https://doi.org/10.1002/ace.7401

Received: September 6, 2023
Accepted: March 28, 2024
Published: August 2, 2024

Copyright © 2024 Tang et al. This is an open-access publication distributed under the terms of the Creative Commons Attribution license.
